# Identifying membrane-bound transcriptional regulatory proteins from rare but evolutionarily conserved domain combinations

**DOI:** 10.1093/nar/gkag635

**Published:** 2026-06-25

**Authors:** Muyoung Lee, Yeejin Jang, Qingqing Guo, Ujwal Punyamurtula, Joonhyuk Choi, Jonghwan Kim, Edward M Marcotte

**Affiliations:** Department of Molecular Biosciences, The University of Texas at Austin, Austin, TX 78712, United States; Department of Molecular Biosciences, The University of Texas at Austin, Austin, TX 78712, United States; Department of Molecular Biosciences, The University of Texas at Austin, Austin, TX 78712, United States; Department of Molecular Biosciences, The University of Texas at Austin, Austin, TX 78712, United States; Department of Molecular Biosciences, The University of Texas at Austin, Austin, TX 78712, United States; Department of Molecular Biosciences, The University of Texas at Austin, Austin, TX 78712, United States; Department of Molecular Biosciences, The University of Texas at Austin, Austin, TX 78712, United States

## Abstract

Transcriptional regulatory proteins, including transcription factors (TFs) and chromatin modifiers, must act inside cell nuclei, but membrane-bound transcription factors (MBTFs) are first anchored in membranes before nuclear translocation. Known MBTFs are vital for processes from myelin expression (MYRF) to cholesterol homeostasis (SREBP), yet their overall diversity remains uncharted. We hypothesized that additional membrane-bound transcriptional regulators (MBTRs) might exist, so we developed a bioinformatics screen to prioritize membrane proteins that are likely to regulate transcription. Our approach leverages domain composition by positing that unusual domain combinations suggest novel biological functions. We searched for rare but evolutionarily conserved pairings of transmembrane domains with domains likely involved in transcriptional regulation. Our method rediscovered known MBTFs and membrane-bound histone kinases, and identified novel MBTR candidates, including transmembrane histone N-acetyltransferases, a putative new subclass of MBTFs that shares the DNA-binding domain of MYRF, and the prolactin regulatory element-binding protein PREB (SEC12). Assays using recombinant PREB demonstrated that the transmembrane domain is the determinant of PREB subcellular localization, governing its distribution between the membrane and the nucleus in mouse embryonic stem cells. These findings underscore the utility of our method and provide a framework for investigating MBTRs that likely facilitate the integration of extracellular signals with transcriptional responses.

## Graphical Abstract

**Graphical Abstract:**
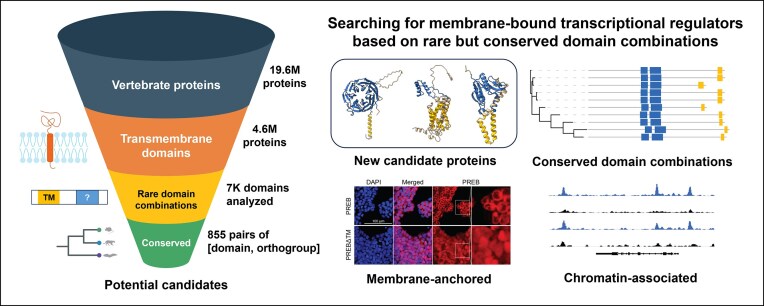

## Introduction

Transcription factors (TFs) regulate transcription by binding to DNA in the nucleus [1], so many TFs are imported into the nucleus from the cytosol. However, some TFs are anchored to membranes by transmembrane domains before their nuclear translocation. These rare TFs, known as membrane-bound transcription factors (MBTFs), play crucial biological functions. For example, SREBP (sterol regulatory element-binding protein) regulates lipid metabolism and homeostasis [2], MYRF (myelin regulatory factor) regulates myelin production by glial cells [3, 4], and ATF6 (activating transcription factor 6) reacts to the accumulation of misfolded proteins in cells [5]. For an MBTF to function as a TF, it must be released from the anchored membrane and transported to the nucleus. Multiple mechanisms are known for this proteolytic release from the transmembrane domains [3, 6, 7].

Efforts to systematically screen for MBTFs have been ongoing, with the first computational prediction of MBTFs published in 2001 [8]. This study identified proteins with selected DNA-binding domains from the Pfam database [9] and transmembrane domains using a consensus of multiple prediction tools. From 20 organisms and one virus, they identified 76 proteins, including six human proteins such as SREBP1, SREBP2, and DMRT2. However, they excluded ATF6 because only two of the four programs predicted its transmembrane domain, and proteins lacking the selected DNA-binding domains were also filtered out. Yao *et al*. identified 1089 MBTFs from a total of 25 850 TFs in 14 plant species, including 64 MBTFs from *Arabidopsis thaliana* [10]. They predicted the presence of transmembrane domains in TFs from PlantTFDB v4.0 [11]. While both studies predicted the existence of transmembrane domains in proteins with selected DNA-binding domains or TFs from a database, the actual number of MBTFs remains largely unknown. Furthermore, it is uncertain how common these types of transcriptional regulation are, including those involving membrane-bound proteins that bind DNA (e.g. MBTFs), modify chromatin, or perform previously undiscovered functions.

In this study, we developed a protein-domain-based search method to identify membrane-bound transcriptional regulators without imposing any specific precondition on the target domain characteristics. Here, we use the term membrane–bound transcriptional regulators (MBTRs) in a broad, operational sense to denote membrane proteins whose cleaved or translocated fragments enter the nucleus and are required for transcriptional or chromatin-state changes. This definition encompasses: (i) classical MBTFs, such as SREBP, NOTCH, and ATF6, whose released fragments function within sequence–specific DNA–bound transcription factor complexes [12–15]; (ii) membrane–anchored chromatin modifiers, including histone kinases and acetyltransferases (for example, TRPM6/7), whose released catalytic domains act on chromatin [16–18]; and (iii) membrane–derived transcriptional co–regulators whose nuclear fragments are necessary for transcriptional responses but whose intrinsic DNA–binding activity is not yet clearly defined.

Many proteins consist of structurally and functionally independent domains that play key roles in determining a protein’s overall function. We hypothesized that by identifying proteins with domains that are prevalent in non-membrane proteins yet scarce in membrane proteins, we might uncover new MBTRs. Given the importance of known MBTFs in vertebrate embryonic development and health, we focused exclusively on vertebrate proteins, aiming to identify new conserved human and mouse MBTRs in the current study, although the algorithm will also generalize to other clades. To identify such conserved proteins, we considered orthologous protein groups (orthogroups) on the hypothesis that orthologous proteins frequently retain similar domain compositions, at least within a limited taxonomic range, such as vertebrates [19, 20]. This trend enables us to investigate whether rare domain compositions are nonetheless conserved across vertebrates within specific orthogroups. We thus developed our search method based on two criteria: (i) domains that rarely pair with transmembrane domains across vertebrate proteins, and (ii) orthogroups in which the domain combination of interest occurs more frequently.

By focusing on rare but evolutionarily conserved combinations of transmembrane domains with transcription-related domains, we rediscovered well-known MBTFs, including SREBP, NOTCH, and ATF6, as well as other MBTRs that have been primarily studied for different biological functions. More importantly, we identified lesser-characterized transmembrane proteins with potential roles in gene regulation. One example is PREB (prolactin regulatory element-binding protein), which we experimentally validated as a potential MBTF. Our results suggested that PREB should undergo proteolytic cleavage, and in the absence of its transmembrane domain, PREB exhibited nuclear translocation and DNA binding activity. Additionally, we identified a potential MBTF class with the same DNA-binding domain as MYRF, which extends beyond vertebrates. We observed that this DNA-binding domain frequently pairs with transmembrane domains across vertebrates, prompting us to expand our analysis to all eukaryotic proteins that possess this domain, identifying a related but distinct candidate MBTR family in fungi. Our findings suggest that MBTRs could be more conserved than previously predicted, indicating their functional significance. This approach can be used to identify novel regulatory mechanisms across diverse biological contexts and taxonomic groups and can be applied to other protein domain combinations.

## Materials and methods

### Collecting domain annotations of vertebrate proteins and predicting transmembrane domains

We downloaded bulk protein data from UniProt [21] and collected domain annotations from Pfam-A entries [9]. To select vertebrate species and their subspecies, we downloaded the list of all descendants of vertebrates (Taxon ID 7742) from UniProt Taxonomy and chose taxa whose ranks were annotated as species or subspecies. Then, we retained proteins from these vertebrate species and subspecies. As a result, we selected 19 650 569 proteins. We ran TMbed [22] to predict whether these proteins have transmembrane domains. We excluded 1599 proteins whose lengths exceeded 8300 amino acids. We assigned 4 653 349 transmembrane proteins and 14 995 621 non-transmembrane proteins. In total, 99.992% of the selected vertebrate proteins were assigned.

We defined a “transmembrane protein” as any protein with at least one alpha-helical or beta-strand transmembrane amino acid segment predicted by TMbed, whether single-pass or multi-pass. Proteins with only N-terminal signal peptides or glycosylphosphatidylinositol anchors and no predicted transmembrane residues were not counted as transmembrane proteins.

We assessed the sensitivity of TMbed and DeepTMHMM on proteins from the Protein Data Bank of TM proteins (PDBTM) [23], which provides both alpha-helical and beta-strand transmembrane protein sequences in FASTA format. Among 33 241 PDBTM alpha-helix entries, TMbed correctly identified 32 666, and DeepTMHMM identified 31 510 entries (sensitivities of 98.27% and 94.79%, respectively; see Supplementary Table S1). For 1245 beta strand entries, TMbed correctly predicted 1038, and DeepTMHMM predicted 806 entries (83.37% and 64.74% sensitivity, respectively). This result shows that TMbed achieves high sensitivity for both types of transmembrane proteins from PDBTM. We also confirmed that DeepTMHMM gives concordant results for the PREB protein with TMbed.

### Mapping orthologous protein groups (orthogroups)

To define orthologous protein families, we used orthogroups from the eggNOG 5.0 database [24], assigning orthogroups to our protein sequences using eggNOG-mapper v2.1.9 [25]. We filtered the mapping results using two criteria: first, we required that each protein have the same taxonomic annotation in UniProt and eggNOG, or that the UniProt taxon be a subspecies of the eggNOG species. We then removed orthogroups containing proteins from a single species only. In total, we assigned 3 798 707 proteins from 102 species and their 92 subspecies to 27 745 orthogroups.

### Calculation of conservation-to-frequency ratios as a means to prioritize MBTRs

We find domains that rarely coexist with transmembrane domains by measuring the following frequency, referred to as *f_all_*:


\begin{eqnarray*}
{\mathrm{Across\ all\ proteins}},{\mathrm{\ for\ each\ domain\ }}X,\\&&{{f}_{all}} = \frac{{n\left( {\textit{transmembrane}{\mathrm{\ }}\textit{proteins}{\mathrm{\ }}\textit{with}{\mathrm{\ }}\textit{domain}{\mathrm{\ }}X} \right)}}{{n\left( {\textit{total}{\mathrm{\ }}\textit{proteins}{\mathrm{\ }}\textit{with}{\mathrm{\ }}\textit{domain}{\mathrm{\ }}X} \right)}},
\end{eqnarray*}

which was expected to be low, given our goal of identifying domains rarely found in a transmembrane context.

To identify domain combinations conserved across vertebrate species, we calculated the same ratio but for each orthogroup, referred to as *f_OG_*:


\begin{eqnarray*}&&{\mathrm{In\ an\ orthogroup}},{\mathrm{\ for\ each\ domain\ X}},\\&&{{f}_{OG}} = \frac{{{\mathrm{n}}( {\textit{transmembrane}{\mathrm{\ }}\textit{proteins}{\mathrm{\ }}\textit{with}{\mathrm{\ }}\textit{domain}{\mathrm{\ }}X{\mathrm{\ }}in{\mathrm{\ }}\textit{orthogroup}} ){\mathrm{\ }}}}{{n( {\textit{total}{\mathrm{\ }}\textit{proteins}{\mathrm{\ }}\textit{with}{\mathrm{\ }}\textit{domain}{\mathrm{\ }}X{\mathrm{\ }}in{\mathrm{\ }}\textit{orthogroup}} )}},\end{eqnarray*}

which was expected to be high for the case of well-conserved domain combinations.

Then, we divided *f_OG_* by *f_all_*, resulting in a list of conservation-to-frequency ratios (*f_OG_*/*f_all_*), one for each [domain, orthogroup] pair. Domains that do not combine with transmembrane domains were excluded, and [domain, orthogroup] pairs with fewer than 10 transmembrane proteins were also filtered out. From the resulting distribution of conservation-to-frequency ratios, 1239 [domain, orthogroup] pairs exhibited values exceeding 1.995 (${{10}^{0.3}}$). To assess whether this heuristic cutoff was statistically justified, we generated a null distribution of conservation-to-frequency ratios by repeatedly permuting protein–orthogroup assignments 10 000 times while preserving orthogroup sizes, then recomputing *f_OG_*/*f_all_* with the same filters. In this null, the 95th and 99th percentiles of *f_OG_*/*f_all_* were 1.27 and 1.64, respectively. This implies that <1% of [domain, orthogroup] pairs would be expected to reach our cutoff (∼1.995) by chance. Based on this null, the estimated false discovery rate (FDR) at our discovery threshold *f_OG_*/*f_all_* ≥ 10^0.3^ was ~2.38%. This FDR refers to the discovery of protein orthogroups with unusual domain combinations, but is not restricted to MBTRs.

Lastly, we selected 855 [domain, orthogroup] pairs that included at least one human or mouse protein for further analysis. (The source code and related resource files for calculating conservation-to-frequency ratios are available for download at https://doi.org/10.5281/zenodo.17351617.)

### Building phylogenetic trees with domain composition visualization

For SREBP1, NOTCH1, ATF6B, PREB, CERS2, and NAT8F3, we aligned their orthologous proteins (here, defined by assignment to the same pre-computed eggNOG orthogroup) using MAFFT v7.505 [26], and inferred approximately maximum-likelihood phylogenetic trees using FastTree v2.1.11 [27]. We selected the 10 most representative orthologs that have both transmembrane domains and domains of interest, using PARNAS v0.1.6 [28], prioritizing reviewed proteins (Swiss-Prot in UniProt). Pfam domains with their positions (location within a protein) were annotated using InterProScan v5.75 [29]. For visualizing phylogenetic trees and domains, we used the iTOL online tool [30]. For proteins with “NDT80/PhoG-like DNA-binding family” domain and transmembrane domains, we downloaded sequences of all proteins with that domain annotation and selected proteins with transmembrane domains by using TMbed. We filtered out proteins with either the “MYRF ICA domain” or the “MYRF C-terminal domain 2” annotation from InterProScan. Subsequently, we constructed the phylogenetic tree and visualized it using iTOL as described earlier.

### Cloning of PREB and PREBΔTM

In order to create the modified construct pSBFB, the luciferase open reading frame (ORF) from the pSBtet-GP vector (Addgene, 60495) was replaced with a FLAG tag, a biotinylation site, and multiple cloning sites containing NotI and NheI restriction sites as previously described [31]. Mouse *Preb* cDNA ORF clones were obtained from Sino Biological (MG5A1071-CY) and amplified using primers designed to produce constructs encoding PREB with or without the transmembrane domain (PREBΔTM) (Supplementary Table S2), with the incorporation of NotI and NheI restriction sites (NEB, R3189, and R3131). The amplified products were cloned into NotI/NheI-digested pSBFB vector using T4 DNA ligase (NEB, M0202). The cloned constructs were verified by Sanger sequencing and transfected into J1 mouse embryonic stem cells (ESCs) stably expressing BirA using Lipofectamine Stem Transfection Reagent (Thermo Fisher Scientific, STEM00008) along with pCMV(CAT)T7-SB100 (Addgene, 34879) for genomic integration. Transfected cells were selected with puromycin (1 µg/ml, Gibco A1113803) and G418 (250 µg/ml, Gibco 10 131 027) starting 24 h after transfection to generate stable cell lines. To induce expression of PREB and PREBΔTM, cells were treated with 0.5 µg/ml of doxycycline (Dox; Fisher BioReagents, BP26535) for 24 h, and protein expression of both constructs was confirmed by western blotting.

### Cell culture

J1 mouse ESCs were cultured on plates coated with 0.1% gelatin in standard ESC culture medium. The medium consisted of Dulbecco’s modified Eagle’s medium (DMEM; Gibco, 11965118), supplemented with 18% fetal bovine serum (FBS; MilliporeSigma, 12306C), EmbryoMax nucleosides (MilliporeSigma, ES-008-D), MEM non-essential amino acids (Gibco, 11-140-050), 0.1 mM β-mercaptoethanol (MilliporeSigma, M3148-25ML), 50 U/ml penicillin–streptomycin–glutamine (PSG, Gibco, 10378016), and 1000 U/ml recombinant mouse leukemia inhibitory factor (LIF; Gemini Bio-Products, 400-495). Cells were maintained at 37°C in a humidified incubator with 5% CO₂ and passaged every 2–3 days using 0.25% trypsin–EDTA (Thermo Fisher Scientific, 25200056) for single-cell dissociation.

H9 human ESCs (WiCell) were cultured on plates coated with Matrigel (Corning, 354234) in mTeSR Plus medium (Stemcell Technologies, 100-0276). Cells were maintained at 37°C in a humidified incubator with 5% CO₂ and passaged every 4–5 days using ReLeSR (Stemcell Technologies, 100-0483).

CT27 human trophoblast stem cells (TSCs; kindly provided by Dr Takahiro Arima, Tohoku University) were cultured as previously described [32]. The cells were cultured on plates coated with 0.05% iMatrix-511 (Iwai North America, N-892012). The medium consisted of DMEM/F12 medium (Thermo Fisher Scientific, 11320082) supplemented with 0.15% BSA (MilliporeSigma, A7906), 0.6% penicillin–streptomycin (Thermo Fisher Scientific, 15140-122), 1% ITS-X supplement (Thermo Fisher Scientific, 5150056), 1% KnockOut Serum Replacement (Thermo Fisher Scientific, 10828028), 0.2 mM L-ascorbic acid (Wako, 013-12061), 2.5 µM Y27632 (Selleck Chemicals, S1049), 25 ng/ml EGF (PeproTech, AF-100-15), 0.8 mM VPA (Wako, 227-01071), 2 µM CHIR99021 (Selleck Chemicals, S2924), and 5 μM A83-01 (MilliporeSigma, SML0788). Cells were maintained at 37°C in a humidified incubator with 5% CO₂ and passaged every 3–4 days with TrypLE Select Enzyme (Thermo Fisher Scientific, A1217701).

Cell collection for mass spectrometry was conducted as follows. H9 and CT27 were washed three times with ice-cold PBS. The cells were then scraped off the plates with a cell lifter (Corning, 3008) and then centrifuged (5 min, 4226 × *g*, 4°C). The remaining PBS supernatant was aspirated, and the pellet was kept at −80°C until protein analysis.

### Western blot

PREB- and PREBΔTM-expressing mouse ESCs, treated with Dox for 24 h, were lysed in Laemmli sample buffer (Bio-Rad, 1610747) supplemented with 5% β-mercaptoethanol (MilliporeSigma, M3148-25ML) and then heated at 95°C for 10 min to denature proteins. Lysates were resolved by 10% sodium dodecyl sulfate–polyacrylamide gel electrophoresis, followed by transfer onto polyvinylidene fluoride (PVDF) membranes (MilliporeSigma, IPVH00010). Membranes were blocked with 5% skim milk in TBS-T (Tris-buffered saline containing 0.1% Tween-20) for 1 h to prevent non-specific binding. Subsequently, the membranes were incubated overnight at 4°C with Streptavidin-HRP (Cytiva, RPN1231-2ML, 1:2000) to detect biotinylated PREB. β-actin antibody (Abgent, AM1829B, 1:20 000) was used as a loading control. After three 10-min washes in TBS-T, membranes for β-actin detection were incubated with an anti-mouse HRP-conjugated secondary antibody (Cell Signaling Technology, 7076, 1:10 000) for 1 h at room temperature. Following another set of three 10-min washes with TBS-T, proteins were detected using enhanced chemiluminescence (ECL) reagent (Cytiva, 45-002-401).

### Immunofluorescence assays

PREB- and PREBΔTM-expressing mouse ESCs, with or without Dox treatment for 24 h, were fixed in 4% paraformaldehyde for 20 min at room temperature, followed by three washes with PBS containing calcium and magnesium (PBS-CM; Corning, 21-040-CV) supplemented with 0.1% bovine serum albumin (BSA; MilliporeSigma, A7906-500G). Following fixation, cells were permeabilized and blocked for 45 min at room temperature in PBS-CM/0.1% BSA containing 0.3% Triton X-100 (MilliporeSigma, T8787-50ML) and 10% horse serum (Gibco, 16050122). Cells were then incubated overnight at 4°C with Streptavidin conjugated to Alexa Fluor 555 (Thermo Fisher Scientific, S21381) in PBS-CM containing 0.1% BSA and 10% horse serum. The next day, cells were washed three times with PBS-CM/0.1% BSA, and nuclei were stained with 4′,6-diamidino-2-phenylindole (DAPI) for 10 min. After final washes, samples were imaged using a Nikon W1 spinning-disk confocal microscope. Identical imaging settings were applied to all samples within each experiment to ensure consistency.

### bioChIP-sequencing experiment

bioChIP-seq was performed on PREB- and PREBΔTM-expressing mouse ESCs treated with 0.5 µg/ml Dox for 24 h as previously described [33]. Cells were cross-linked with 1% formaldehyde (Fisher Bioreagents, BP531-500) for 7 min at room temperature, and the reaction was quenched by adding glycine (Fisher Scientific, BP381-1) to a final concentration of 0.125 M. The chromatin was sheared to an average fragment size of ∼200 bp using a Bioruptor sonicator (Diagenode). Cross-linked chromatin was pre-cleared with Protein A agarose beads (Thermofisher, 10008D) and subsequently subjected to precipitation using Dynabeads MyOne Streptavidin T1 (Invitrogen, 65602). Precipitated complexes were washed twice with 2% SDS, once with buffer A (0.1% deoxycholate, 1% Triton X-100, 1 mM EDTA, 50 mM HEPES pH 7.5, 500 mM NaCl), once with buffer B (250 mM LiCl, 0.5% NP-40, 0.5% deoxycholate, 1 mM EDTA, 10 mM Tris-Cl pH 8.1), and twice with TE buffer. The crosslinked protein–DNA complexes in the elution buffer (1% SDS, 10 mM EDTA, 50 mM Tris-Cl pH 8) were incubated at 65°C overnight for reverse crosslinking. RNase A (Thermo Scientific, EN0531) was then treated for 30 min, followed by treatment of proteinase K (NEB, P8107S) for 2 h. DNA was extracted with phenol:chloroform:isoamyl alcohol (Invitrogen, 15593031), then precipitated and re-suspended in the TE buffer. Library preparation was performed using the NEBNext Ultra DNA Library Prep Kit (NEB, 7103L), and sequencing was conducted on an Illumina NovaSeq 6000 platform to generate 50-bp paired-end reads. Comparison of the samples with the BirA-only control allowed identification of target-specific binding sites, and IgG ChIP served as an additional control.

### bioChIP-sequencing analysis

The ChIP-seq raw DNA sequencing data were adapter-trimmed using TrimGalore v0.6.10 (https://github.com/FelixKrueger/TrimGalore), aligned by Bowtie v2.5.1 [34], and then processed with Samtools v1.20 [35] and Picard MarkDuplicates v3.0.0 (http://broadinstitute.github.io/picard). Peak calling was performed using Macs3 v3.0.1 [36], and the heatmap was generated using DeepTools v3.5.5 [37]. Consensus peaks shared by at least 3 replicates were identified using bedtools intersect v2.31.1 [38]. Motif enrichment analysis was performed by HOMER2 v5.1 [39].

To classify PREBΔTM-binding sites based on chromatin accessibility, bioChIP-seq peaks from PREBΔTM-expressing mouse ESCs following Dox induction were compared with accessibility profiles from wild-type ESCs using publicly available ATAC-seq data (GEO accession GSE220724) [31]. ATAC-seq signal over PREB-binding loci was quantified and visualized using DeepTools. Motif enrichment analysis was performed on PREBΔTM-specific peaks using HOMER2.

### Site-1 protease and Site-2 protease inhibitor treatment

PREB- and PREBΔTM-expressing mouse ESCs were seeded one day prior to treatment. The following day, cells were treated with 0.5 µg/ml Dox to induce expression, together with the S1P inhibitor PF-429242 (Selleck Chemicals, S6418) or the S2P inhibitor nelfinavir mesylate (Selleck Chemicals, S4282) at 10 µM. Cells were maintained under these conditions for 24 h and 48 h to assess the effects on proteolytic cleavage. DMSO was used as the control.

### Chromatin fractionation of cell lysates

The chromatin precipitation workflow was modified from Shiio *et al*. [40] as follows: harvested human ESC (H9) and human TSC (CT27) pellets were lysed by resuspending in Pierce IP lysis buffer (3:1 v/v ratio of lysis buffer to sample; 25 mM Tris–HCl, pH 7.4, 150 mM NaCl, 1 mM EDTA, 1% NP-40, and 5% glycerol; Thermo Fisher) containing 1× cOmplete mini EDTA-free protease inhibitor (Roche) and 1× PhosSTOP EASYpack phosphatase inhibitor (Roche). These resuspended samples were incubated on ice for 5 min and vortexed periodically. To help separate subcellular components into insoluble and soluble fractions, the resulting cell lysates were homogenized using a 7-ml KIMBLE Dounce tissue grinder set (Millipore) with 5 passes of pestle A and 45 passes of pestle B. To further clarify insoluble and soluble components, the homogenized cell lysates were flash-frozen with liquid nitrogen and thawed immediately by hand. Finally, to obtain separated insoluble and soluble fractions, the cell lysates were centrifuged at 14 000 × *g* for 20 min at 4°C. The pellet fraction samples (for both cell types) were resuspended in 100 μl of Pierce IP lysis buffer and subjected to another freeze-thaw cycle to break apart insoluble material before flash-freezing for overnight storage.

### Mass spectrometry sample preparation

Chromatin-fractionated cell lysates were prepared for mass spectrometry analysis using the combinative sample preparation strategy [41]. To solubilize the samples, SDS was added to 2% of the final volume, after which samples were heated to 95°C for 15 min. Subsequently, samples were diluted in a 6:1 acetone/sample volume ratio with ice-cold acetone (previously cooled to −20°C) and incubated overnight (∼15 h) at 4°C. Acetone-treated samples were centrifuged (16 600 × *g*, 15 min, 4°C), and the supernatant was discarded. Sample precipitates were washed twice with 400 μl acetone (16 000 × *g*, 20 min, 4°C). To solubilize the proteins in the precipitate (and enable in-solution digestion), sample precipitates were resuspended in 50 μl 1% SDC/50 mM NH_4_HCO_3_, and sonicated twice for 10 min. Solubilized proteins were reduced with TCEP (final concentration: 5 mM) at 56°C for 45 min and then alkylated with 25 mM IAM for 45 min in the dark at RT. After quenching unreacted IAM with 12 mM DTT, proteins were digested overnight (∼15 h at 37°C) with 2 μg proteomics-grade trypsin. After quenching the tryptic digestion with 1% formic acid, samples were centrifuged (16 000 × *g*, 10 min, 4°C) to remove the precipitated SDC. Samples were then filtered (14 000 × *g*, 20 min, 4°C) using Amicon Ultra 10 kDa filters (Millipore) and dried *via* speed-vacuum centrifugation for 1 h. Samples were then processed using 6 μg C18 ZipTip pipette tips (Millipore), eluted, dried once more by speed vacuum centrifugation, and resuspended in 5% acetonitrile/0.1% formic acid for LC-MS/MS analysis.

### Mass spectrometry data acquisition

Peptides were separated by reverse phase chromatography using a Dionex Ultimate 3000 RSLCnano UHPLC system (Thermo Scientific) on an EASY-Spray PepMap RSLC C18 column (Thermo Scientific, ES902). Peptides were eluted using a 3%–45% acetonitrile gradient in 0.1% formic acid over 120 min. The mass spectra of LC-separated peptides were acquired on a Thermo Orbitrap Fusion Lumos mass spectrometer using a data-independent acquisition method adapted from Federation *et al*., with some modifications [42]. The Fusion Lumos was configured to acquire DIA spectra using 42 × 12 m/z sequential precursor isolation windows at 15 000 resolution across a precursor mass range of 400–900 m/z. After each pass through the precursor mass range (i.e. after 42 DIA MS/MS scans), a full precursor MS scan was performed (400–1600 m/z, 120 000 resolution, AGC target 4e5).

### Mass spectrometry data analysis

DIA MS/MS experiments were searched using the DIA-NN platform [43], with a predicted spectral library generated from a Uniprot protein FASTA file for *Homo sapiens*. The DIA-NN search utilized standard settings (trypsin digestion with up to one missed cleavage, deep learning-based spectra/RT/IM prediction, fixed cysteine carbamidomethylation, N-terminal methionine excision, and a precursor FDR of 1%). For each subcellular fraction in each cell type (i.e. ESC insoluble, ESC soluble, TSC insoluble, and TSC soluble), three technical replicates were collected.

## Results

### A search for conserved domains that are found rarely in transmembrane proteins suggests new membrane-bound transcriptional regulators

**Figure 1.**
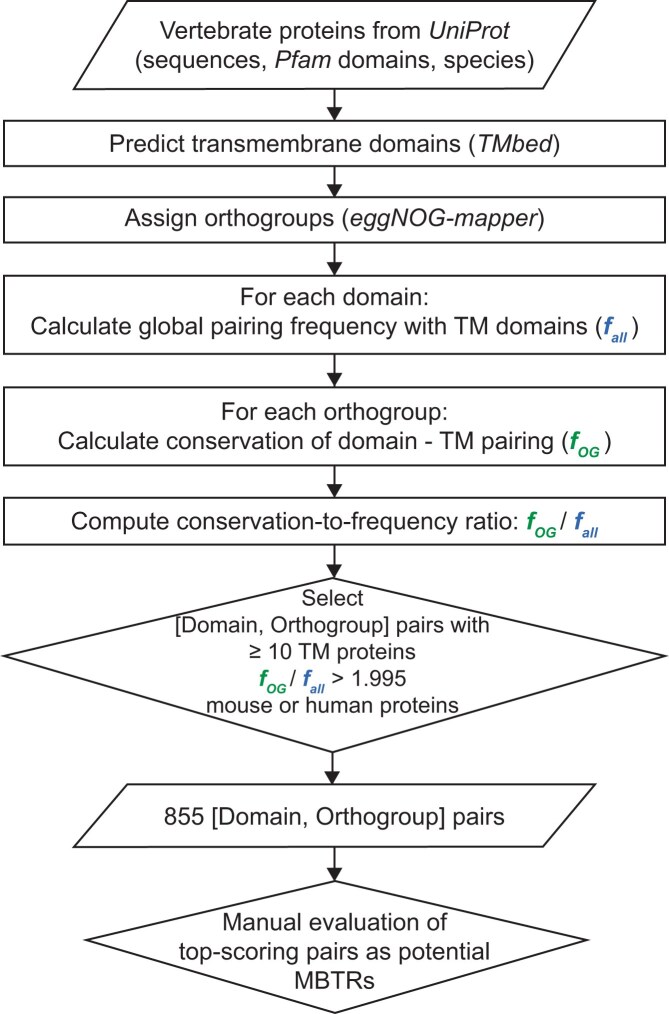
Search method to identify conserved protein domains that are rarely found in a transmembrane context as a means to discover potential new membrane-bound regulatory proteins. Our method aims to find proteins satisfying the following two conditions: (i) having a domain that rarely pairs with transmembrane domains, but for which (ii) the combination of the domain of interest and transmembrane domain is nonetheless well-conserved in some orthogroups. The ratios corresponding to these two conditions were calculated and represented by *f_all_* and *f_OG_* values, respectively. Then, we calculated the conservation-to-frequency ratios (*f_OG_*/*f_all_*) for [domain, orthogroup] pairs. After filtering out [domain, orthogroup] pairs with annotated conditions, we investigated proteins in the final list.

In this study, we aimed to discover new MBTRs by developing a search method for protein domains that rarely pair with transmembrane domains, but, when found, are well-conserved across species, focusing here on vertebrate MBTRs. To develop the search method, we first collected information on vertebrate proteins, including their domain and orthogroup annotations, as well as the presence of transmembrane domains. We downloaded amino acid sequences and Pfam domain annotations [9] of 19.65 million (M) vertebrate proteins from UniProt [21]. Transmembrane domains were predicted based on sequences using TMbed [22], resulting in 4.65 M transmembrane proteins and 15 M non-transmembrane proteins. To investigate evolutionary conservation, we transferred the eggNOG orthogroup annotations to our proteins. As a result, 27 745 orthogroups encompassing 3.8 M proteins from 102 vertebrate species were assigned and used in the subsequent sections of this study (Fig. 1).

To find domains that rarely pair with transmembrane domains, we calculated the frequency of transmembrane proteins for each domain across all proteins (*f_all_*; Fig. 1). If this value is low for a domain, it indicates that the domain and transmembrane domain rarely coexist in vertebrate proteins. Then, to check whether that domain combination is nonetheless conserved within specific orthogroups, we calculated the frequency of transmembrane proteins for each domain within each orthogroup (*f_OG_*; Fig. 1). Since orthologous proteins can be highly conserved and can have highly similar domain combinations, the *f_OG_* value can be correspondingly high in some orthogroups even if the overall *f_all_* is low. We then considered both the global frequency and the conservation of domain combinations by calculating the ratio of these frequencies (*f_OG_*/*f_all_*; Fig. 1). We defined this ratio as the conservation-to-frequency ratio.

Some domains are widely distributed in membrane receptors, which could bias the domain counts. To assess this, we examined both the global frequency *f_all_* and orthogroup-specific frequency *f_OG_*. For example, the “Calcium-binding EGF domain” (PF07645) has a high *f_all_* (0.348), so even for an orthogroup with a high *f_OG_* value, the resulting conservation-to-frequency ratios remain modest. In addition, we counted whether a domain is present in a protein, rather than how many copies it has, so repeated copies do not inflate *f_OG_* values.

**Figure 2.**
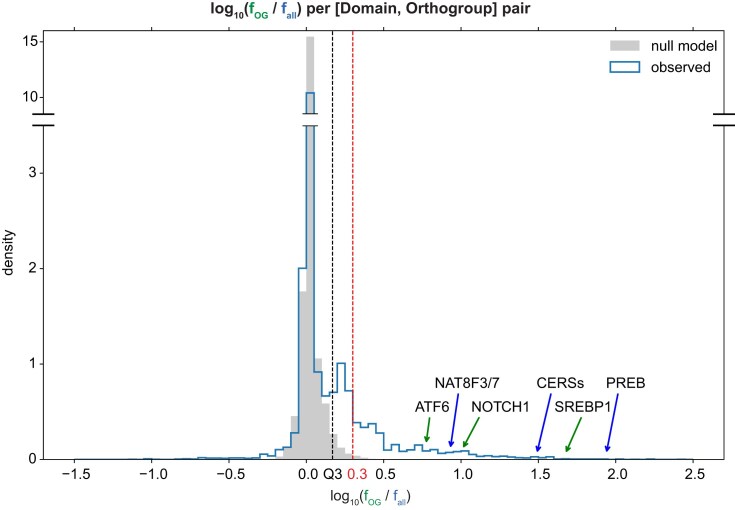
Known membrane-bound transcriptional regulators exhibit high conservation-to-frequency ratios (*f_OG_*/*f_all_*), accompanied by new candidate regulators. The distribution of the logarithm base 10 of the conservation-to-frequency ratios for [domain, orthogroup] pairs with at least 10 transmembrane proteins (Q1: 0.0, Median: 0.0018, Q3: 0.1692) compared with the null distribution model, which was obtained by permuting protein–orthogroup assignments. Based on this distribution, we selected a heuristic cutoff of 1.995 (≅10^0.3^). Three known MBTRs and three new candidate MBTRs are labeled. (We annotate only Q3 because Q1 and the median are very close to zero, and marking all three values would introduce visual clutter.)

From this analysis, we made a list of all [domain, orthogroup] pairs with their corresponding conservation-to-frequency ratios. To remove spuriously large values coming from small orthogroups, we filtered out [domain, orthogroup] pairs with fewer than 10 transmembrane proteins. Then, based on the distribution of conservation-to-frequency ratios, we selected a heuristic cutoff (*f_OG_*/*f_all_* = 1.995) for discovery (Fig. 2). Since there is no curated set of MBTRs, formal benchmarking (precision, recall, and FDR) against reference sets was not available. Instead, we generated a null distribution by permuting orthogroups, which implied that few [domain, orthogroup] pairs would reach our cutoff by chance. The false discovery rate for the discovery of unusual domain combinations was estimated to be 2.38%. We additionally excluded [domain, orthogroup] pairs that contained no human or mouse proteins, yielding 855 [domain, orthogroup] pairs (Supplementary Table S3) and manually examined the top-scoring families.

### Well-known MBTFs were detected by our method

**Figure 3.**
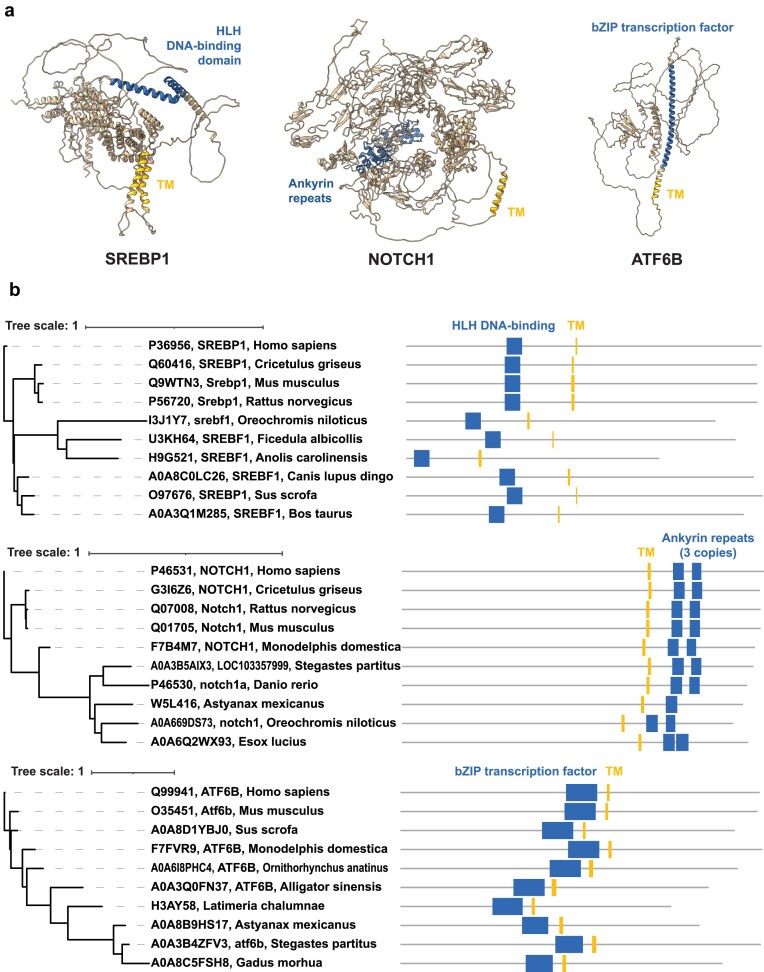
Predicted structures and phylogenetic trees of orthologs illustrate the conserved domain compositions for three well-known MBTFs with high conservation-to-frequency ratios. (**a**) AlphaFold predictions of three well-known MBTFs based on mouse protein sequences. Transmembrane domains are marked in yellow. The domains that comprise the combination with transmembrane domains are colored blue. (**b**) Phylogenetic trees showing 10 orthologs of each MBTF with their Pfam domain annotations (top: SREBP1, middle: NOTCH1, bottom: ATF6B). The same color scheme as panel (a) was applied.

We first verified our method by assessing whether previously known MBTFs were detected. Our results included several well-known MBTFs: SREBP1 (sterol regulatory element-binding protein 1), SREBP2, NOTCH1 (neurogenic locus notch homolog protein 1), NOTCH2, NOTCH3, NOTCH4, ATF6 (cyclic AMP-dependent transcription factor ATF-6 alpha), ATF6B, CREB3L1 (cyclic AMP-responsive element-binding protein 3-like protein 1), CREB3L3, and CREB3L4 (Table 1). Figure 3a shows predicted structures of SREBP1, NOTCH1, and ATF6B from AlphaFold [44]. The phylogenetic trees constructed from orthologs of these proteins illustrate the conservation of specific domain pairs across several vertebrate species (Fig. 3b). Both the conservation of domains at the orthogroup level and the AlphaFold structural predictions of alpha-helical transmembrane domains are in concordance with these proteins being correctly classified by conservation-to-frequency ratios, suggesting this general workflow will also be valid for investigating subsequent new predictions.

**Table 1.** Well-known MBTFs detected by our method

Representative proteins	Pfam ID	Domain	*f_all_*	eggNOG ID	Orthogroup	*f_OG_*	*f_OG_*/*f_all_*
ATF6	PF00170	bZIP transcription factor	0.16	ENOG50497SM	transcription factor	0.96	5.93
ATF6B	PF00170	bZIP transcription factor	0.16	ENOG5048W9C	activating transcription factor 6 beta	0.96	5.90
CREB3L1	PF00170	bZIP transcription factor	0.16	ENOG50490X8	cAMP responsive element binding protein 3-like 1	0.76	4.69
CREB3L3	PF00170	bZIP transcription factor	0.16	ENOG50496Y8	cAMP response element binding	0.73	4.49
CREB3L4	PF00170	bZIP transcription factor	0.16	ENOG5049MMU	cAMP response element binding	1.00	6.18
	PF00170	bZIP transcription factor	0.16	ENOG5049074	cAMP response element binding	0.73	4.53
NOTCH1	PF12796	Ankyrin repeats (3 copies)	0.10	ENOG5048WDE	neurogenic locus notch homolog protein	0.97	10.14
	PF07645	Calcium-binding EGF domain	0.35	ENOG5048WDE	neurogenic locus notch homolog protein	0.91	2.63
NOTCH2	PF13857	Ankyrin repeats (many copies)	0.09	ENOG5048V3W	neurogenic locus notch homolog protein	0.96	10.16
	PF12796	Ankyrin repeats (3 copies)	0.10	ENOG5048V3W	neurogenic locus notch homolog protein	0.92	9.69
	PF07645	Calcium-binding EGF domain	0.35	ENOG5048V3W	neurogenic locus notch homolog protein	0.80	2.28
NOTCH3	PF13857	Ankyrin repeats (many copies)	0.09	ENOG5049EBI	glomerular capillary formation	0.96	10.22
	PF12796	Ankyrin repeats (3 copies)	0.10	ENOG5049EBI	glomerular capillary formation	0.95	10.02
	PF07645	Calcium-binding EGF domain	0.35	ENOG5049EBI	glomerular capillary formation	0.92	2.65
NOTCH4	PF00023	Ankyrin repeat	0.07	ENOG50497TI	Neurogenic locus notch homolog protein 4	0.99	14.32
	PF13637	Ankyrin repeats (many copies)	0.08	ENOG50497TI	Neurogenic locus notch homolog protein 4	0.96	12.12
	PF12796	Ankyrin repeats (3 copies)	0.10	ENOG50497TI	Neurogenic locus notch homolog protein 4	0.94	9.89
	PF07645	Calcium-binding EGF domain	0.35	ENOG50497TI	Neurogenic locus notch homolog protein 4	0.89	2.56
SREBP1	PF00010	Helix-loop-helix DNA-binding domain	0.01	ENOG50495AZ	sterol response element binding	0.58	46.93
SREBP2	PF00010	Helix-loop-helix DNA-binding domain	0.01	ENOG50494W8	sterol regulatory element binding transcription factor 2	0.16	12.74

SREBP1 had a conservation-to-frequency ratio of 46.93, ranking 10th overall, and SREBP2’s value, 12.74, also falls within the top 10%. Both proteins regulate lipid homeostasis and cholesterol synthesis [45], and their “helix-loop-helix DNA-binding domain” was flagged by our approach. This domain exhibited a very low *f_all_* value of 0.01, indicating that it rarely pairs with transmembrane domains, except across members of their corresponding “sterol response element binding” and “sterol regulatory element binding transcription factor 2” orthogroups. Interestingly, SREBP1 and SREBP2 had low *f_OG_* values compared to other top-scoring protein families (0.58 and 0.16, respectively), but since their *f_all_* values were even lower, they scored well in our analysis. Thus, the combination of “helix-loop-helix DNA-binding domain” and transmembrane domains is somewhat less conserved, but this combination is even more scarce, and our approach is still able to assign a high score to this known MBTF family.

Different from SREBPs, all four NOTCH proteins were detected multiple times by different [domain, orthogroup] pairs with conservation-to-frequency ratios from 2.28 to 14.32. Our method flagged NOTCH proteins’ “ankyrin repeats” and “calcium-binding EGF domain” domains. The ankyrin repeat is one of the most common protein–protein interaction motifs and is found widely in proteins with diverse functions. The “calcium-binding EGF domain” was named after the epidermal growth factor, where it was first described. This domain can be found in many membrane-bound and extracellular proteins [46], and our results showed that its *f_all_* value is higher than that of ankyrin repeats. Orthogroups for NOTCH1, NOTCH2, and NOTCH4 were explicitly related to NOTCH homologs, while NOTCH3 belongs to a “glomerular capillary formation” orthogroup, consistent with its known role in maintaining the structural integrity and functional stability of renal arteries [47]. These orthogroups had high *f_OG_* values, indicating that the combination of domains (of interest and transmembrane domain) was well conserved. Since NOTCH proteins are transmembrane receptors, this result was plausible.

The third protein group included ATF6 and ATF6B. Their conservation-to-frequency ratios were 5.93 and 5.90, respectively. These proteins are involved in the endoplasmic reticulum (ER) stress response, specifically the unfolded protein response. Under ER stress, the N-terminal cytoplasmic domain is cleaved and translocates to the nucleus [5, 48]. The domain flagged by our method was “bZIP transcription factor,” which has the basic region and the leucine zipper region. Its *f_all_* value was 0.16, relatively low. Although the orthogroup of ATF6 is rather generically named “transcription factor,” almost all members of this orthogroup appear to correspond to ATF6 from various species. ATF6B’s orthogroup, “activating transcription factor 6 beta,” was also particular to ATF6B. These two orthogroups had high *f_OG_* values, indicating that these proteins exhibit well-conserved, rare domain combinations.

The last protein group with the lowest conservation-to-frequency ratios among well-known MBTFs consisted of three CREB3L (cyclic AMP-responsive element-binding protein 3-like) proteins: CREB3L1 (4.69), CREB3L3 (4.49), and CREB3L4 (6.18 and 4.53). CREB3L1 responds to ER stress or DNA damage, activating genes that inhibit cell cycle progression. CREB3L3 is proposed to act during ER stress and may function with ATF6. CREB3L4 may also contribute to the unfolded protein response [49–51]. Our method flagged the “bZIP transcription factor” domain, whose *f_all_* value was 0.16. Orthogroups of these three proteins were “cAMP responsive element binding protein 3-like 1” and “cAMP response element binding,” and *f_OG_* values were around 0.75, except for one small orthogroup (ENOG5049MMU). Those *f_OG_* values indicate that some CREB3L orthologous proteins lack the transmembrane domain.

### Our method rediscovered MBTRs that have been mainly studied for other biological functions

**Figure 4.**
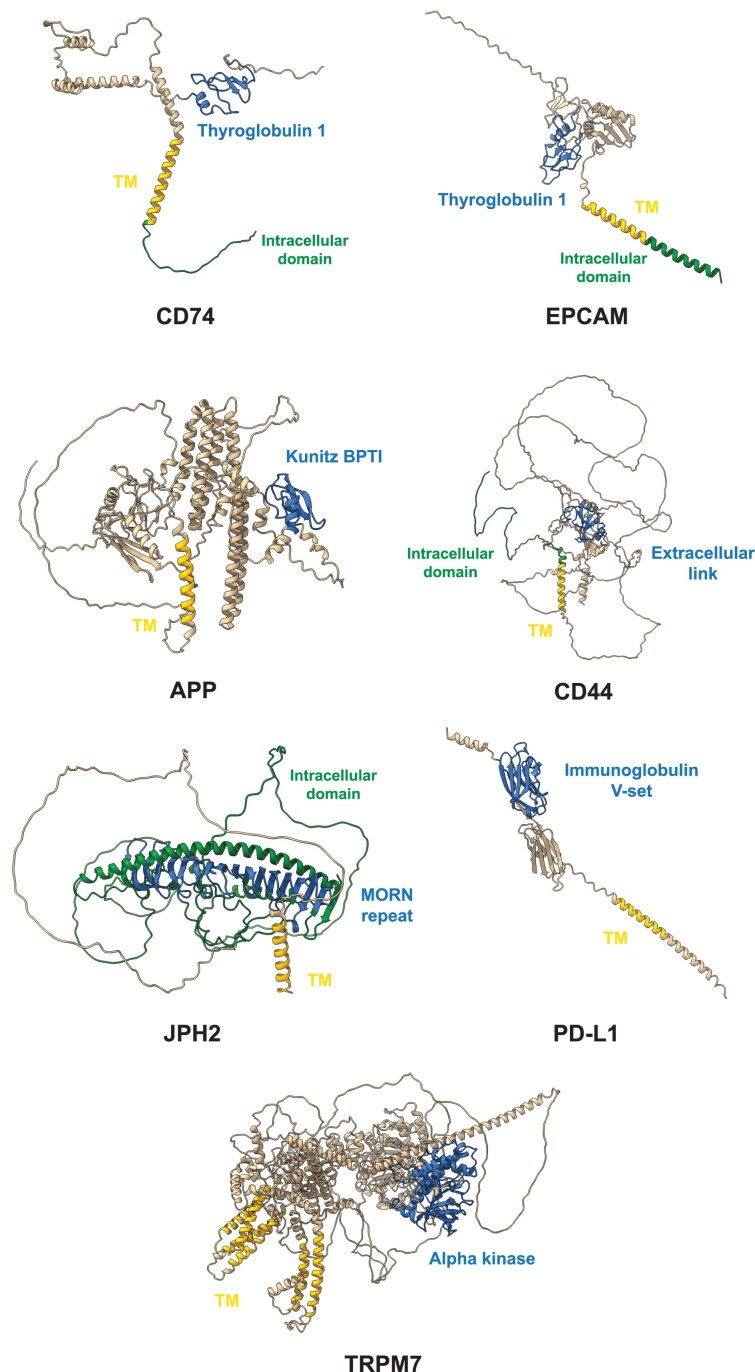
Predicted structures with domain annotations of MBTRs that have been mainly studied for their non-transcriptional-regulatory functions. AlphaFold-predicted structures of MBTRs captured by our method that have primarily been studied for their non-transcriptional-regulatory biological functions. Transmembrane domains are marked in yellow, the domains that pair with transmembrane domains are marked blue, and cytoplasmic intracellular domains are colored in green if available. (The structure of LRP1 was excluded due to its massive size, exceeding 4500 amino acids).

After validating that our search method can find well-known MBTFs, we asked if other less well-studied MBTFs, as well as the broader category of MBTRs, were scored highly by our search. In particular, we were interested in transmembrane proteins with evidence in the literature for regulating transcription, but for which this role was not necessarily well-characterized or broadly recognized. We identified nine MBTRs (Table 2; Fig. 4) that employ four distinct activation mechanisms, demonstrating the diversity of membrane-to-nucleus signaling. While their conservation-to-frequency ratios are generally modest compared to canonical MBTFs, their discovery underscores our method’s sensitivity in detecting regulatory proteins. (Since LRP1 is a large protein over 4500 amino acids, its structure prediction is not included in Fig. 4).

**Table 2.** Characterized MBTRs rediscovered by our search that have been mainly studied for other biological functions

Category	Representative proteins	Pfam ID	Domain	*f_all_*	eggNOG ID	Orthogroup	*f_OG_*	*f_OG_*/*f_all_*
**RIP** (regulated intramembrane proteolysis)-dependent	CD74	PF00086	Thyroglobulin type-1 repeat	0.13	ENOG50494AH	MHC class II protein binding, *via* antigen binding groove	0.98	7.56
	EPCAM	PF00086	Thyroglobulin type-1 repeat	0.13	ENOG50491FU	Epithelial cell adhesion molecule	0.96	7.39
	LRP1	PF12662	Complement Clr-like EGF-like	0.19	ENOG5048WNX	alpha-2 macroglobulin receptor activity	0.77	4.06
		PF07645	Calcium-binding EGF domain	0.35	ENOG5048WNX	alpha-2 macroglobulin receptor activity	0.80	2.30
	APP	PF00014	Kunitz/Bovine pancreatic trypsin inhibitor domain	0.31	ENOG50494FN	Amyloid beta (A4)	0.93	3.03
	CD44	PF00193	Extracellular link domain	0.38	ENOG504993T	CD44 molecule (Indian blood group)	0.91	2.40
**Stress-responsive** proteolytic release	JPH2	PF02493	MORN repeat	0.16	ENOG50490UH	phosphatidylinositol-5-phosphate binding	0.62	3.73
**Non-proteolytic** nuclear translocation	PD-L1 (CD274)	PF07686	Immunoglobulin V-set domain	0.32	ENOG5048WVQ	Programmed cell death 1 ligand	0.86	2.66
**Enzymatically active** domain release	TRPM6, TRPM7	PF02816	Alpha-kinase family	0.39	ENOG5048X4Y	calcium-dependent cell-matrix adhesion	0.97	2.48

The largest group (5 proteins: CD74, EPCAM, LRP1, APP, and CD44) is activated through regulated intramembrane proteolysis (RIP), a conserved mechanism in which sequential proteolytic cleavages release an intracellular domain (ICD) that translocates to the nucleus [52–59]. CD74 and EPCAM exhibit the highest conservation-to-frequency ratios (7.56 and 7.39) among the nine rediscovered MBTRs. CD74 releases an intracellular domain (CD74-ICD) that modulates genes involved in apoptosis, immune response, and cell migration through interactions with RUNX and NF-κB [53]. Similarly, the cleavage of EPCAM generates the EPCAM intracellular domain (EP-ICD), which drives genes involved in cell division, pluripotency, and EMT-associated processes [60]. LRP1, APP, and CD44 showed lower values (4.06/2.30, 3.03, and 2.40) but share a similar architecture. LRP1-ICD co-activates PPARγ, a central regulator of lipid and glucose metabolism [61]. APP’s ICD regulates multiple genes, including KAI1, Neprilysin, and p53 [57]. CD44-ICD activates matrix metalloproteinases [62].

The second activation mechanism employs a stress-induced proteolytic cleavage, exemplified by JPH2, a membrane protein cleaved in cardiomyocytes under mechanical stress. This stress-induced cleavage generates an N-terminal fragment that translocates to the nucleus and activates cardioprotective genes, demonstrating how structural and transcriptional reprogramming are coordinated in the stressed heart [63]. PD-L1 demonstrates an alternative activation mechanism. Although it is primarily observed in cancer cells, deacetylation of PD-L1 by HDAC2 triggers its nuclear translocation without cleavage, where it regulates immune-response genes [64]. This case demonstrates that the membrane anchoring can be controlled epigenetically, not always through irreversible proteolytic cleavage. TRPM6 and TRPM7 (conservation-to-frequency ratio: 2.48) expand the MBTRs’ functional scope beyond TFs. They are ion channels but contain a serine/threonine kinase at their carboxyl termini. Upon cleavage, their C-terminal kinase domains translocate to the nucleus and phosphorylate histones, thereby directly modifying chromatin structure [16–18].

Together, these nine proteins illustrate the mechanistic diversity underlying membrane-bound transcriptional regulation, encompassing canonical RIP, stress-induced cleavage, non-proteolytic nuclear translocation, and cleavage-dependent release of histone kinase. Evolution has thus repeatedly used switchable membrane anchoring as a key regulatory component across a variety of biological processes. Importantly, these examples illustrate that our approach is agnostic as to the specific mechanism employed or the particular domains used, hence opening the possibility for discovering new classes of MBTRs.

### New candidate MBTRs include PREB, ceramide synthases, and N-acetyltransferases

**Figure 5.**
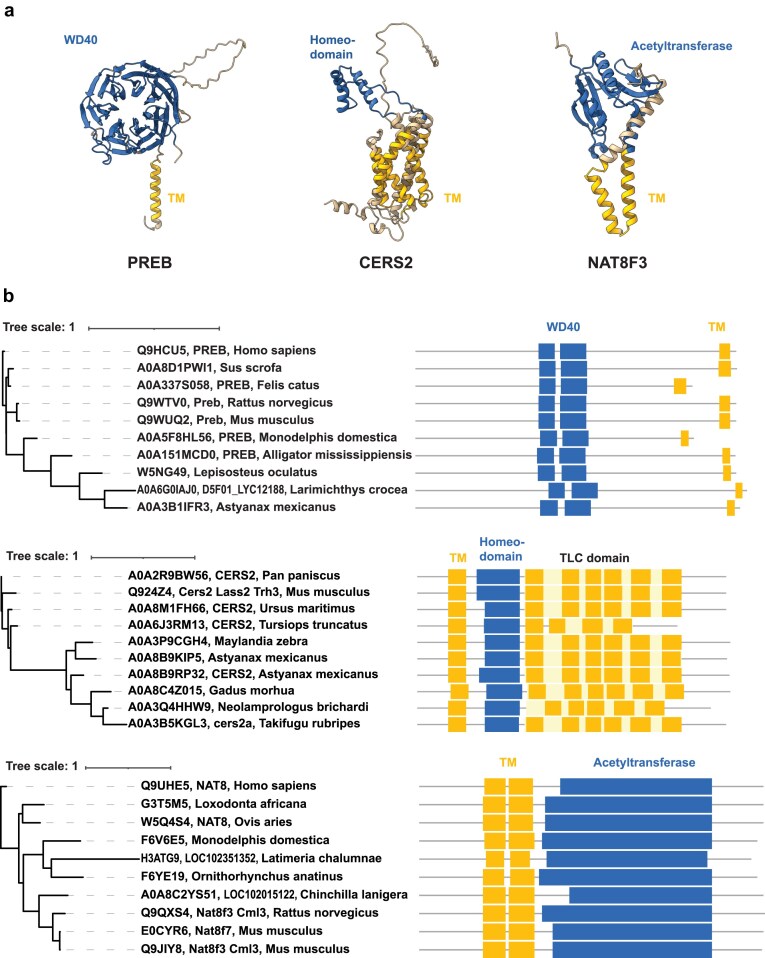
Based on their conserved domain compositions, PREB, CERS2, and NAT8F3 are new candidate MBTRs. (**a**) AlphaFold-predicted structures for three potential MBTRs, here showing the mouse proteins. Transmembrane domains are marked in yellow. The domains that comprise the combination with the transmembrane domain are colored blue. (**b**) Phylogenetic trees showing 10 orthologs of each candidate protein with their Pfam domain annotations (top: PREB, middle: CERS2, bottom: NAT8F3 and NAT8F7). The TLC domain includes internal transmembrane domains. The same color scheme as panel (a) was applied.

Having confirmed that our search method could identify both already-known MBTFs and MBTRs more generally, we next searched for novel candidate MBTRs amongst the high-scoring protein families (Table 3; Fig. 5).

**Table 3.** New candidate MBTRs

Representative proteins	Pfam ID	Domain	*f_all_*	eggNOG ID	Orthogroup	*f_OG_*	*f_OG_*/*f_all_*
PREB	PF00400	WD domain, G-beta repeat	0.01	ENOG504999H	prolactin regulatory	0.84	86.57
CERS2	PF00046	Homeodomain	0.03	ENOG5048YJT	regulation of Schwann cell proliferation involved in axon regeneration	1.00	30.76
CERS3	PF00046	Homeodomain	0.03	ENOG50497XU	sphingosine N-acyltransferase activity	1.00	30.76
CERS4	PF00046	Homeodomain	0.03	ENOG5048X03	sphingosine N-acyltransferase activity	1.00	30.76
CERS5, CERS6	PF00046	Homeodomain	0.03	ENOG5048VWA	sphingosine N-acyltransferase activity	1.00	30.76
NAT8F3, NAT8F7	PF00583	Acetyltransferase (GNAT) family	0.12	ENOG5049MJF	N-acetyltransferase	0.98	8.50

PREB (prolactin regulatory element-binding protein) was ranked one of the highest-scoring protein families of our entire analysis. It has a “WD domain, G-beta repeat” domain annotation with the *f_all_* value 0.01, one of the lowest values in the results. WD repeat domains are known to interact with diverse binding partners, such as proteins, DNA, and RNA [65]. PREB’s WD repeat domain forms a seven-bladed beta-propeller structure, with a short alpha-helix transmembrane domain on the C-terminal side (Fig. 5a). PREB’s orthogroup annotation was “prolactin regulatory” with an *f_OG_* value of 0.84, mostly consisting of PREB proteins from various species. The phylogenetic tree of 10 PREB orthologs shows conservation of the WD and transmembrane domains across vertebrates (Fig. 5b).

The next candidates in our results were ceramide synthases (CERSs): CERS2, CERS3, CERS4, CERS5, and CERS6, which have multiple transmembrane domains. Ceramide synthases catalyze the transfer of the acyl chain from acyl-CoA to a sphingoid base, and six isoforms of mammalian CERS, except CERS1, contain a homeodomain [66]. Our results recovered the homeodomain annotation for these CERS proteins (PF00046; *f_all_*: 0.03; Fig. 5a). The corresponding orthogroups (“sphingosine N-acyltransferase activity,” “regulation of Schwann cell proliferation involved in axon regeneration,” *f_OG_* values were 1.00 for both), were highly CERS-specific. The phylogenetic tree of 10 CERS2 orthologs shows the conservation of both the homeodomain and transmembrane domains across several vertebrate species (Fig. 5b). It was reported that Schlank, a Drosophila CERS, binds to DNA *via* its homeodomain, and mouse CERS2 can also regulate transcription [67]. However, human CERS2 lacks residues critical for DNA binding, making it unclear whether the homeodomain’s binding function has been conserved. Therefore, although we suggest CERSs as evolutionarily motivated MBTR candidates based on conserved domain combinations, their direct participation in DNA binding and transcriptional regulation remains hypothetical and requires experimental validation.

Another interesting case is NAT8F3 (N-acetyltransferase family 8 member 3) and NAT8F7, mouse proteins reported to be histone acetyltransferases that prefer histone H4 and exhibit perinuclear localization. It was suggested that these proteins are conserved across chordates but originated in cnidaria [68]. Their domain annotation was “acetyltransferase (GNAT) family” (PF00583; *f_all_*: 0.12; Fig. 5a), and the orthogroup was “N-acetyltransferase” (*f_OG_*: 0.98). The phylogenetic tree of 10 orthologs shows both acetyltransferase domains and transmembrane domains (Fig. 5b). While the extent to which these histone acetyltransferase activities are conserved across vertebrates is currently unknown, our analysis suggests that this family could be a new MBTR, along with PREB and the ceramide synthases.

In order to provide more direct experimental support for this activity, we next focused our effort on evaluating the top-scoring PREB family.

### PREB without its transmembrane domain suggests a potential processing event, nuclear translocation, and DNA binding

PREB was historically identified as a DNA-binding protein that regulates prolactin, a hormone responsible for lactation [69]. It was subsequently reported that PREB also binds to the promoters of insulin and adiponectin, and regulates gluconeogenic genes [70–72]. Therefore, PREB’s function appeared from these data to be predominantly related to lactation, glucose, and lipid metabolism. However, there are two apparently largely disjoint sets of literature observations on PREB, which is also alternatively known as Sec12, a guanine nucleotide exchange factor that plays a crucial role in the formation of coat protein complex II (COPII) vesicles [73] and, moreover, is far more widespread in expression than its historic name would suggest. COPII vesicles transport proteins from the ER to the Golgi apparatus. For this function, PREB should be anchored on the membrane. In contrast, as a TF, PREB should enter the nucleus. These two bodies of literature suggest that the PREB protein has a dual functionality. Considering PREB as a candidate MBTR would thus imply that the membrane-bound form is responsible for regulating vesicle formation, while a proteolytically released cytoplasmic domain might translocate to the nucleus to regulate transcription.

**Figure 6.**
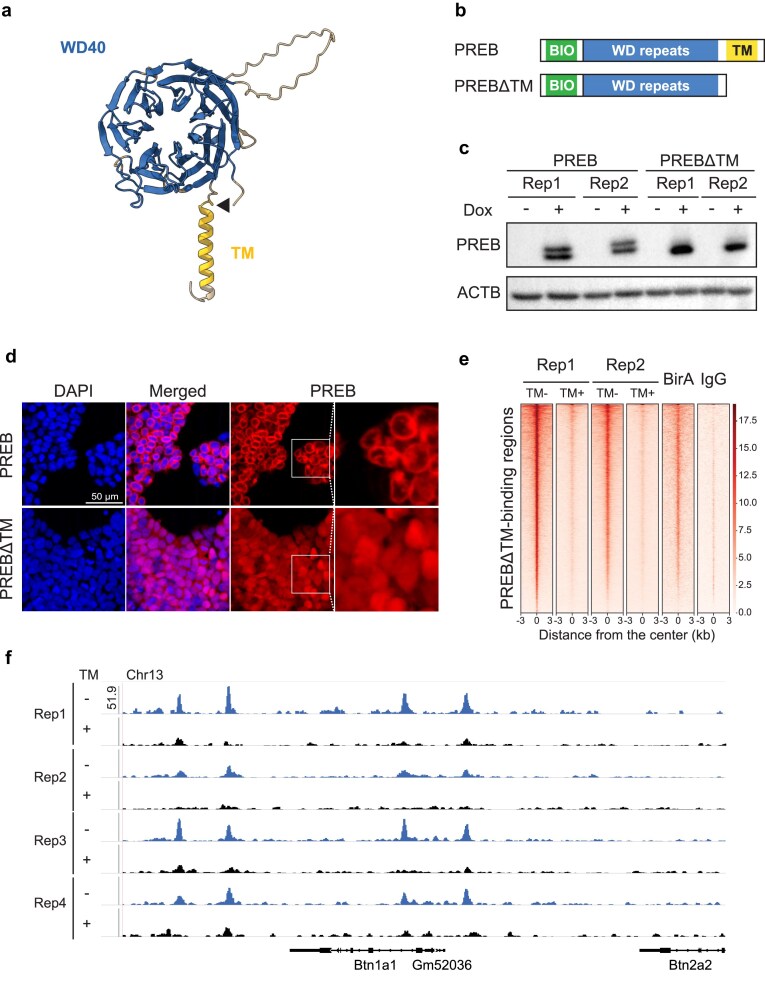
PREB without its transmembrane domain undergoes nuclear translocation and binds to the butyrophilin locus. (**a**) Predicted structure of PREB generated by AlphaFold, highlighting the WD repeat domains, the transmembrane domain, and the site of an engineered C-terminal truncation. (**b**) Schematic of the two constructs created to assay the mouse full-length PREB protein and PREBΔTM, lacking the C-terminal transmembrane domain. The N-terminal biotinylation site is indicated. (**c**) Western blot results of two biological replicates of mouse ESCs expressing PREB and PREBΔTM. Two bands for PREB were detected by streptavidin–HRP, suggesting C-terminal processing of a full-length PREB when expressed in mouse ESCs. (**d**) Immunofluorescence confocal microscopy images of PREB and PREBΔTM following Dox induction. PREB localizes predominantly to a perinuclear region, forming a ring-like shape. PREBΔTM displays diffused nuclear localization. Rightmost panels show zoomed images corresponding to the boxed regions. (**e**) bioChIP-seq profiles for PREB, PREBΔTM, BirA-only streptavidin pulldown, and IgG ChIP-seq control samples. PREBΔTM samples show substantially stronger DNA-binding activity than full-length PREB. (**f**) Representative locus-specific binding profiles of PREB and PREBΔTM at the Btn1a1 (butyrophilin subfamily 1 member A1) gene. PREBΔTM shows markedly higher peak intensities, consistent with enhanced nuclear localization in the absence of the transmembrane domain.

To investigate whether PREB lacking its transmembrane domain (PREBΔTM) translocates to the nucleus and binds DNA, we ectopically expressed cDNAs for full-length PREB and PREBΔTM in mouse ESCs using the pSBFB Dox-inducible overexpression system [33, 74, 75] (Fig. 6a and b). Dox induction did not elicit detectable morphological changes for both cases (Supplementary Fig. S1). However, western blot analysis revealed multiple protein bands following induction of full-length PREB, whereas PREBΔTM appeared as a single band (Fig. 6c), suggesting potential proteolytic processing of the full-length protein. Notably, as the expression clone was derived from a cDNA ORF, we can rule out alternative splicing in this context. Because the pSBFB vector introduces an N-terminal tag detectable by streptavidin-HRP, the presence of the tag in both bands indicates that processing occurs at the C-terminus (Fig. 6c).

Immunofluorescence imaging showed a perinuclear ring for full-length PREB consistent with ER proximity, while PREBΔTM displayed a predominant nuclear signal by DAPI overlap (Fig. 6d), supporting the hypothesis that the transmembrane domain prevents nuclear translocation. Given the nuclear localization of PREBΔTM, we hypothesized that it may exhibit stronger DNA-binding activity than PREB. To test this, we performed biotin-mediated chromatin immunoprecipitation followed by sequencing (bioChIP-seq) upon ectopic expression of PREB and PREBΔTM. To confirm the specificity of the binding events, we used a BirA-only control to check endogenous biotinylation and an IgG control for non-specific association. Aligning with the nuclear localization signal of PREBΔTM, the bioChIP-seq result showed that PREBΔTM exhibited a markedly greater increase in genome-wide DNA binding compared with full-length PREB (Fig. 6e). Based on previous literature [69], we expected PREBΔTM binding peaks near the prolactin gene, but this was not observed in mouse ESCs under the conditions tested. We detected the binding peaks near other genes, most notably the butyrophilin gene (Fig. 6f). Motif enrichment analysis of PREBΔTM ChIP-seq peaks (consensus peaks from ≥3 replicates) revealed strong enrichment for canonical ESC pluripotency TF motifs, including OCT4–SOX2–NANOG, Sox family, Klf, and Esrrb (Supplementary Table S4). This pattern is more consistent with the recruitment of PREB to established ESC regulatory elements than with nonspecific absorption to open chromatin. To further assess the specificity of PREBΔTM binding, we compared PREBΔTM ChIP-seq peaks with ATAC-accessible regions in wild-type ESCs. Only a fraction of ATAC-accessible regions overlapped with PREBΔTM-bound sites, while the majority did not (Supplementary Fig. S2), demonstrating that PREBΔTM does not indiscriminately occupy all open chromatin. Consistent with this, motif enrichment analysis of PREBΔTM-specific peaks also showed enrichment of Esrrb, OCT4–SOX2–TCF–NANOG, and other pluripotency TF motifs (Supplementary Table S5), indicating that the binding pattern is not a trivial consequence of open chromatin accessibility. However, our current data do not distinguish whether PREB recognizes these sites via interactions with other TFs. The future identification of interacting partner proteins (for example, by IP-MS or BioID) will be essential to clarify the mechanisms of its binding.

Although these experiments do not reflect physiological conditions, the results indicate that PREBΔTM can relocate into the nucleus and associate with chromatin, supporting PREB as a candidate MBTF, pending validation of endogenous processing in relevant contexts. We note that immunohistochemistry of endogenous PREB in human appendix, colon, and rectum tissues from Human Protein Atlas [76] does show that PREB can natively localize to the nucleus of a subset of cells in these tissues (Supplementary Fig. S3); unfortunately, no such data exist for the pituitary gland, where PREB was first isolated as a DNA-binding protein, although experiments by Suzuki, Fliss, and co-authors confirmed nuclear localization in the rat pituitary tumor GH3 cell line [69].

Additionally, we attempted to measure whether endogenous PREB is cleaved in a naïve setting by isolating chromatin-associated proteins from a placental cell type (human trophoblast stem cells), as PREB is known to be highly expressed in the placenta, as well as from human ESCs. Using protein mass spectrometry, we observed endogenous PREB in the detergent-insoluble chromatin fraction in both cell types, demonstrating its nuclear localization and general DNA association. This suggests that a significant fraction of PREB in these cells should lack the transmembrane domain, which we assessed by examining the unique tryptic peptides observed in our mass spectrometry experiments. Human PREB is 417 amino acids long, with the transmembrane domain located at residues 389–409; note also that residues 297–339 form a single, large tryptic peptide unlikely to be observed in our experiments due to its high mass. Across all samples of both cell types, we observed 12 unique tryptic peptides, covering 62% of the PREB amino acids from residues 1–294, but none from the C-terminal region, and residues 297–339 are unlikely to be observed (Supplementary Fig. S4). This is indirect evidence for the lack of the transmembrane domain, but given the otherwise relatively high coverage of peptides in PREB’s N-terminus, it is not incompatible, and at least suggestive, of an absent C-terminus.

Lastly, since the proteases responsible for PREB cleavage in ESCs and the precise cleavage site remain unknown, we also investigated whether PREB has a canonical site-1 protease (S1P) motif, a feature shared with multiple other known MBTFs [77], and found matched sequences. Since site-2 protease (S2P) acts in concert with S1P, we treated PREB-expressing mouse ESCs with inhibitors of S1P and S2P (PF-429242 and nelfinavir, respectively [78, 79]). However, at least under the conditions tested, inhibition of these proteases did not detectably alter PREB cleavage (Supplementary Fig. S5).

### Applying the method to a broader range of species, we found a potential MBTF family

Our method found known and novel potential MBTRs, but protein myelin regulatory factor (MYRF), a well-known MBTF, wasn’t rediscovered. We investigated MYRF’s domains and found that, in our method, MYRF scored poorly because its domains frequently pair with transmembrane domains in vertebrates. Notably, one of the domains is a known DNA-binding domain, “NDT80/PhoG-like DNA-binding family,” related to the yeast meiosis-specific transcription factor NDT80 [80]. We wondered whether this specific domain paired frequently with transmembrane domains outside of vertebrates, so we applied our method more broadly to eukaryotic proteins having the “NDT80/PhoG-like DNA-binding family” annotation. As a result, we found three orthogroups with high *f_OG_* values. Two orthogroups contained MYRF and corresponded to MYRF orthologs, with some occurring outside of animals, as for the choanoflagellate *Monosiga* (e.g. the gene A9UP12_MONBE), which lacks myelin. This broad phylogenetic distribution of MYRF orthologs has been well-characterized previously, with MYRF family members pre-dating animals and controlling transcriptional programs unrelated to myelin in these other species, as previously reviewed [81].

**Figure 7.**
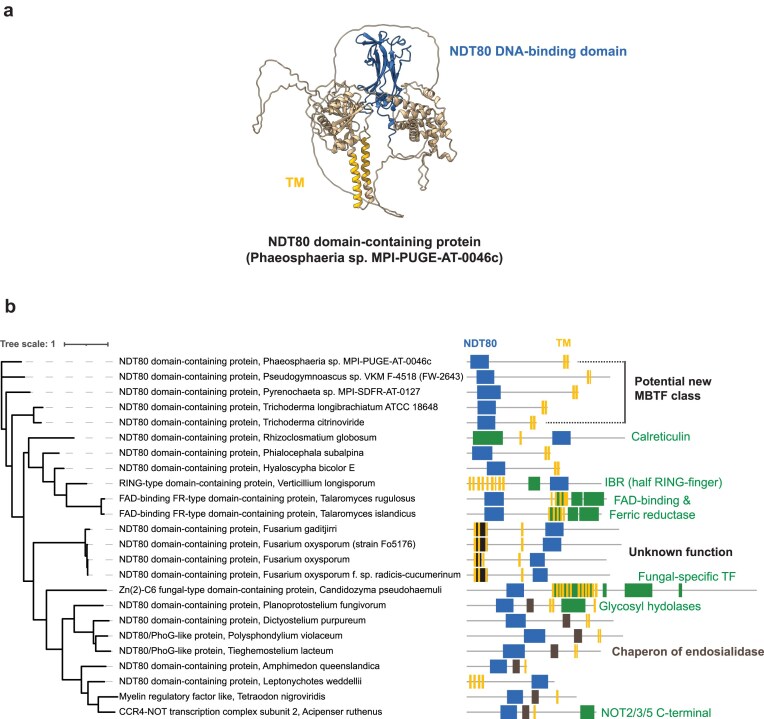
A predicted structure and domain compositions of potential MBTF orthologs containing the NDT80 DNA-binding domain. (**a**) AlphaFold-predicted structure of an NDT80 domain-containing protein (UniProt entry: A0A9P9Q0Z2_9PLEO). Predicted transmembrane domains are marked in yellow, and the NDT80 DNA-binding domains are shown in blue. (**b**) Phylogenetic tree and domain compositions of eukaryotic proteins harboring both the “NDT80/PhoG-like DNA-binding family” domain and transmembrane domains, but lacking MYRF-related domains (“Myelin regulatory factor ICA domain” and “Myelin gene regulatory factor C-terminal domain 2”). The same color scheme as in panel (a) is used, with additional colors (green, brown, and black) for other protein domains. The vertical bar denotes a putative novel fungal MBTF family.

However, in addition, we also identified a small protein orthogroup of unknown function (ENOG502D42H) that does not include MYRF and which may represent a new fungal MBTF class comprising the NDT80 DNA-binding domain with a conserved transmembrane domain, but which lacks MYRF’s signature intramolecular chaperone autoprocessing (ICA) domain (Fig. 7a; see the first five proteins marked with a vertical bar in Fig. 7b). We investigated whether there are more proteins outside of this orthogroup that have both the “NDT80/PhoG-like DNA-binding family” and transmembrane domains, without other typical MYRF-related protein domains (“Myelin regulatory factor ICA domain” and “Myelin gene regulatory factor C-terminal domain 2”) (Fig. 7b). The result includes two new fungal proteins with the same domain composition as the aforementioned small subfamily, which could be additional homologs. Other proteins have additional unrelated domains: Four proteins from the *Fusarium* genus share the same “Protein of unknown function (DUF3176)” domain. Seven proteins from various species (two fish species, one sponge, and four species belonging to Amoebozoa) contain a “chaperone of endosialidase” domain, which is also found in MYRF, but they lack MYRF’s C-terminal domain. Based on the variation even in this one family, we strongly suspect that applying our method to a broader range of species than vertebrates should enable the identification of considerably more MBTRs.

## Discussion

In this study, we developed a novel strategy to identify MBTRs based on protein domain combinations and conserved orthogroups. This approach successfully rediscovered known MBTFs and membrane-bound chromatin modifiers while also revealing candidate MBTRs that had not been previously recognized. We further observed that several MBTFs were initially characterized for biological roles unrelated to transcriptional regulation, with their regulatory roles recognized only later. This trend underscores the utility of our method for identifying multifunctional proteins whose atypical modes of transcriptional regulation have been previously overlooked. Moreover, our findings suggest that MBTRs are conserved across vertebrate species and are even more broadly distributed across eukaryotes, as exemplified by NDT80 DNA-binding domain proteins. These results are consistent with a widespread regulatory paradigm in which extracellular cues are coupled to transcriptional responses within well-represented orthogroups. Quantitative assessment of this prevalence will require expanded orthology resources, systematic sensitivity analyses, and functional validation across diverse taxonomic groups.

Our identification of diverse MBTRs reveals that transcriptional regulation in membrane-bound contexts operates through multiple mechanisms that extend beyond the canonical regulated intramembrane proteolysis (RIP) observed in proteins such as SREBP and ATF6 [3]. Proteins such as PD-L1 demonstrate that membrane anchoring can also be modulated by post-translational modifications, including deacetylation, thereby allowing nuclear translocation without irreversible proteolytic cleavage. The presence of non-proteolytic mechanisms suggests that certain MBTRs may function as reversible transcriptional switches. Furthermore, membrane-bound histone modifiers, such as TRPM6/7, whose kinase domains modify chromatin structure *via* histone phosphorylation, expand the functional repertoire of MBTRs beyond classical TFs. Collectively, the mechanistic diversity highlights membrane anchoring as a versatile regulatory strategy, providing a spatial sequestration of transcriptional regulators until appropriate signals trigger their release. We note that RIP represents the predominant activation mode for MBTRs we identified. Because our method explicitly emphasizes “globally rare but locally conserved” domain combinations, the framework naturally prioritizes RIP-type substrates rather than generic multi-pass receptors bearing common extracellular domains.

Nevertheless, several considerations apply to our search strategy. First, our domain-based framework naturally depends on current domain annotations and transmembrane prediction tools. Second, TMbed, Pfam, and eggNOG were selected for their scalability, and the successful recovery of NOTCH, SREBP, and ATF6 supports their suitability for this screen. Third, when we narrow down [domain, orthogroup] pairs, we applied filters to reduce noise in conservation-to-frequency ratios; however, these criteria may preferentially enrich for larger, well-annotated protein families, and our method does not recover all members of known or suspected MBTR families. For example, all four NOTCH proteins (NOTCH1–4) and five ceramide synthases (CERS2-6) surpass our threshold, whereas CERS1 is not expected to appear because it lacks a homeodomain, which allows other CERS proteins to be detected. In the CREB3 family, CREB3 and CREB3L2 have conservation-to-frequency ratios of 1.989 and 1.228, respectively, which fall below our cutoff, so they are not called by our method. The permutation-derived null distribution implies an FDR of 2.38% at our threshold. Thus, our method is specific for conserved, rare domain-TM combinations but misses some divergent family members, as anticipated. Still, recovery of SREBP1/2, NOTCH 1–4, and ATF6/6B at high conservation-to-frequency ratios supports the utility of our strategy. Fourth, it is possible that predicted transmembrane proteins are trafficked primarily to the extracellular space, lysosomes, or other non-nuclear destinations rather than to the nucleus. Our pipeline does not apply filters beyond predicting the presence of transmembrane domain and rare domain combinations, so such candidates may appear in our results. This is one reason we considered manual inspection of top candidates to be an essential step, and functional experimental validation remains critical for identifying genuine MBTRs. Lastly, candidate rankings can shift with the threshold settings, minimum transmembrane protein filters, and species sampling.

We validated PREB as a candidate MBTF with dual functionality by demonstrating that deletion of its transmembrane domain alters its subcellular localization and enables DNA binding, thereby mimicking the outcome of a potential proteolytic activation mechanism. Future proteomic approaches to identify interaction partners and post-translational modifications will be essential for elucidating the mechanisms underlying its dual roles. This behavior supports a model in which membrane anchoring functions as a molecular switch that controls the spatial and temporal deployment of PREB activity. Such dual functionality might be more widespread than we have acknowledged. The evolution of such bifunctional proteins, which serve as both membrane-bound sensors and transcriptional regulators, represents a simple yet effective solution for coordinating multiple biological processes across cellular compartments. The molecular architecture of MBTRs may offer a practical framework for engineering synthetic regulatory switches that link membrane-associated signals to programmable gene expression responses.

While our *in vitro* experiments with PREB provide compelling evidence for its function as a potential MBTF that undergoes proteolytic cleavage, several limitations warrant consideration. Our studies were conducted in mouse ESCs, human ESCs, and human trophoblast stem cells (TSCs), which may not reflect the physiological context in which PREB exerts its native functions. Previous studies have argued that PREB plays prominent roles in lactation, glucose metabolism, and lipid metabolism, implicating tissues such as the mammary glands and other metabolically active organs as more relevant settings for functional assessments. A visual survey of PREB localization as measured by immunohistochemistry in the Human Protein Atlas [76] reveals mixed cytoplasmic/membrane localization in many cell types assayed, but strong nuclear localization in a subset of cell types, as in the human appendix, colon, and rectum tissues in Supplementary Fig. S3. ChIP-seq experiments in these tissues will also be important for identifying context-specific target genes and refining PREB’s biological roles. Our mass spectrometry experiments with endogenous PREB in human ESCs and TSCs showed that ~62% of the observable amino acids from PREB prior to position 339 were observed by mass spectrometry, but none after, including the TM domain and C-terminal tail. This indirect evidence represents our current best evidence for either processing or alternative splicing, leading to a nuclear-localized, chromatin-bound form of the PREB protein. At this stage, we cannot rule out alternative splicing of the endogenous gene as a means of controlling differential localization, which will require detailed characterization of splice isoforms in cell types showing the differential localization. We also acknowledge that nuclear localization of PREBΔTM could, in principle, arise as a byproduct of protein turnover. However, several independent studies have demonstrated that PREB binds to promoter regions and regulates transcription of genes such as prolactin and adiponectin [69–72], arguing against nuclear PREB being a mere byproduct of protein turnover. Additionally, although an overexpression-based system has been shown to be effective, it may perturb endogenous regulatory mechanisms. While the presence of multiple bands in the western blot suggests proteolytic processing, we did not directly map cleavage products, and alternative post-translational modifications or partial degradation cannot be excluded at this stage. Lastly, our current experiments do not identify a specific upstream signal that dynamically modulates PREB activity. Several prior studies have shown that PREB binds multiple gene promoters and modulates transcriptional activity in cell–type–specific contexts, consistent with a regulatory role. With MYRF, several years elapsed between discovery of its processing mechanism [3, 4] and a mechanism regulating that processing [82, 83]; hence, while we do not identify a specific upstream signal that dynamically modulates PREB activity, it is also not possible to rule out its existence without further study.

The analysis of the NDT80/PhoG-like DNA-binding domain illustrates how our method can uncover potential MBTF families in other eukaryotic lineages when applied beyond vertebrates. The putative fungal protein family we identified shares the core architecture of MYRF—a DNA-binding domain co-occurring with transmembrane domains—yet lacks MYRF’s hallmark ICA domain. This example represents an initial step toward systematically mapping the evolutionary gain and loss events in domain combinations. A comprehensive phylogenomic reconstruction of these events across all MBTR candidate families, including the comparison between vertebrate and invertebrate lineages, will be an important direction for future research.

Finally, although our study focused on proteins containing transmembrane domains in combination with other domains, especially DNA-relevant domains, to identify transmembrane transcriptional regulators, the underlying strategy is readily extensible to other types of regulatory proteins characterized by rare domain combinations. For example, proteins that couple RNA-binding domains with enzymatic domains might be candidates to link metabolic signals and post-transcriptional regulation, whereas metabolic enzymes fused to cytoskeletal proteins could integrate metabolic feedback regulation to cell structural form or integrity. More broadly, our orthogroup-based filtering strategy can also be applied to any domain combination of interest to assess whether such domain combinations are preserved intact over evolution and thus likely to be functionally meaningful. In this way, the approach provides a generalizable way to identify multifunctional proteins that coordinate diverse cellular processes through unexpected combinations of protein domains.

## Supplementary Material

gkag635_Supplemental_Files

## Data Availability

The ChIP-seq data have been submitted to the NCBI Gene Expression Omnibus (GEO; https://www.ncbi.nlm.nih.gov/geo/) under accession number GSE309766. The bigwig track files are viewable in the UCSC Genome Browser session at https://genome.ucsc.edu/s/10myl200/mm10%2C%20PREB%20bioChIP%2Dseq. The source code and related resource files for calculating conservation-to-frequency ratios are available for download at https://doi.org/10.5281/zenodo.17351617. Proteomics data have been deposited in the PRoteomics IDEntifications (PRIDE) database under accession PXD076503.
