# Supplementary material for: Identifying membrane-bound transcriptional regulatory proteins from rare but evolutionarily conserved domain combinations
Source: Nucleic Acids Res. 2026 Jun 25;54(12):gkag635. doi: 10.1093/nar/gkag635 (PMC13294678; doi:10.1093/nar/gkag635)
Supplement: gkag635_Supplemental_Files [file gkag635_supplemental_files.zip › Supplementary_material.docx]

# Supplementary Figures


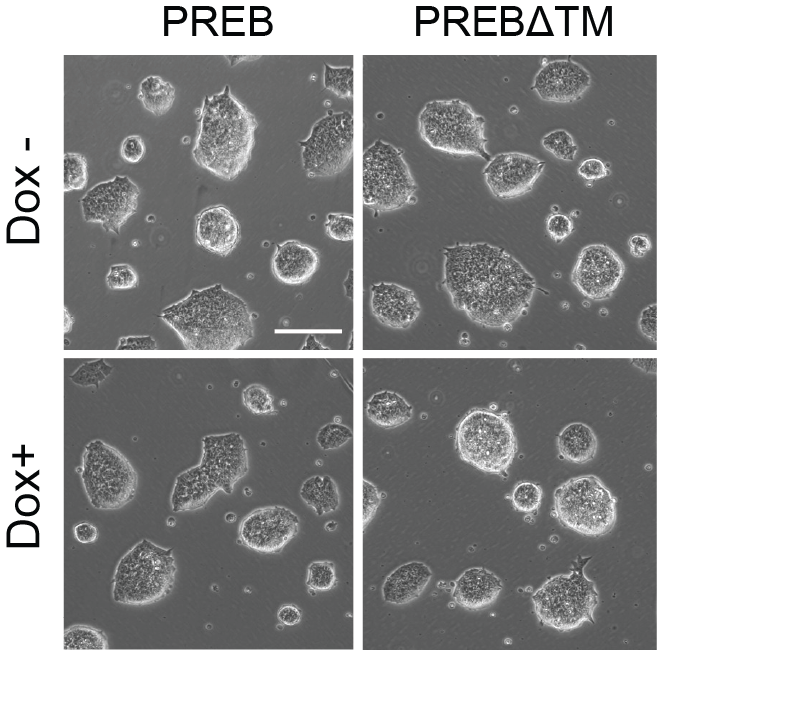


## Supplementary Figure S1. Overexpression of PREB does not change the morphology of mouse embryonic stem cells

Brightfield images of mouse embryonic stem cells expressing full-length PREB and PREBΔTM following treatment with 0.5 µg/mL of Dox for 24 hours (Dox+). Untreated cells (Dox-) were used as controls (Scale bar: 100 µm).


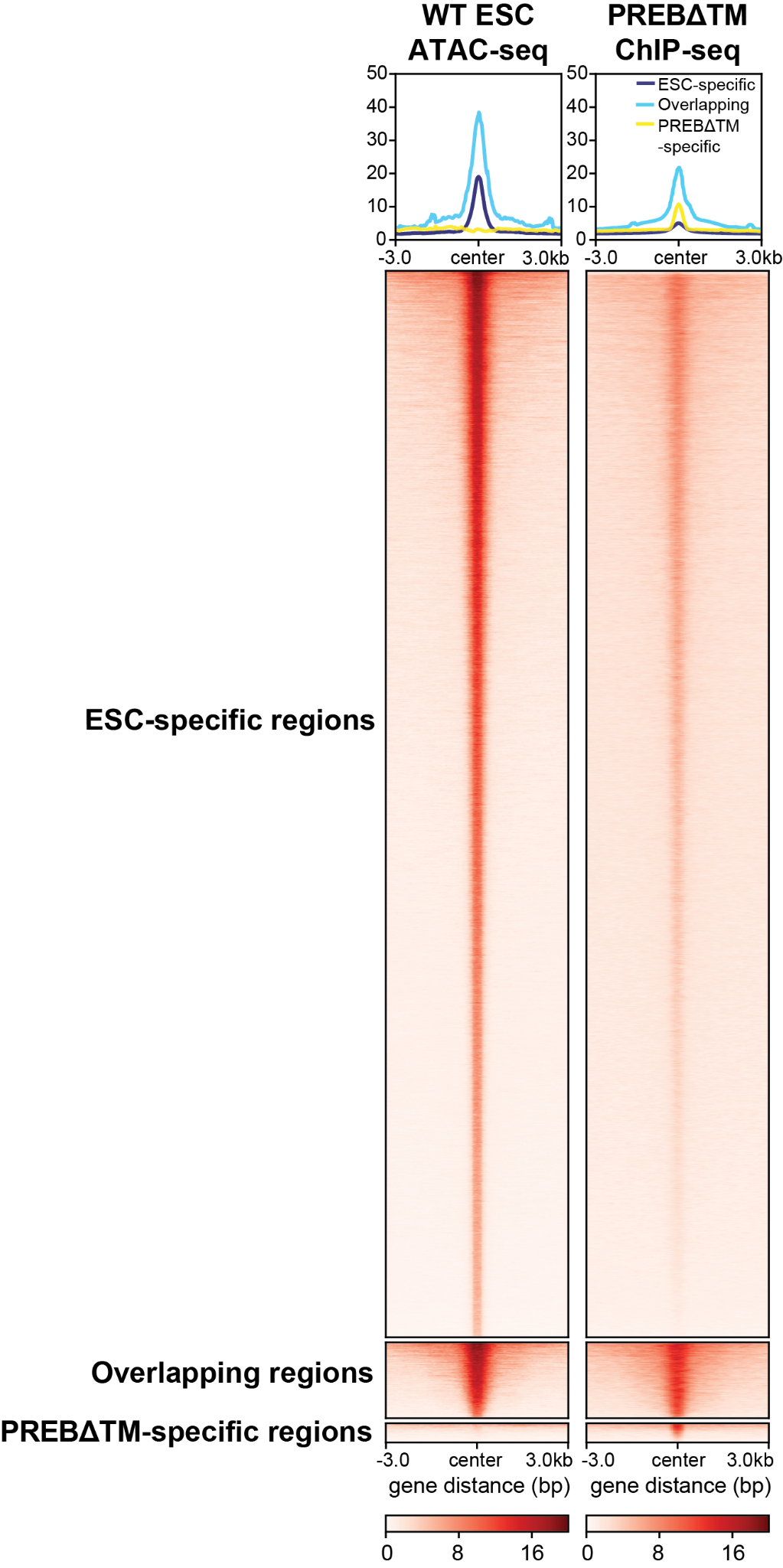


## Supplementary Figure S2. PREBΔTM ChIP-seq peaks share only a fraction of ATAC-accessible regions in wildtype mouse embryonic stem cells

Of the total ATAC-accessible regions in wildtype (WT) mouse embryonic stem cells (ESCs), only a fraction overlapped with PREBΔTM ChIP peaks (overlapping regions), while the majority did not (ESC-specific regions), demonstrating selectivity of PREBΔTM.

#
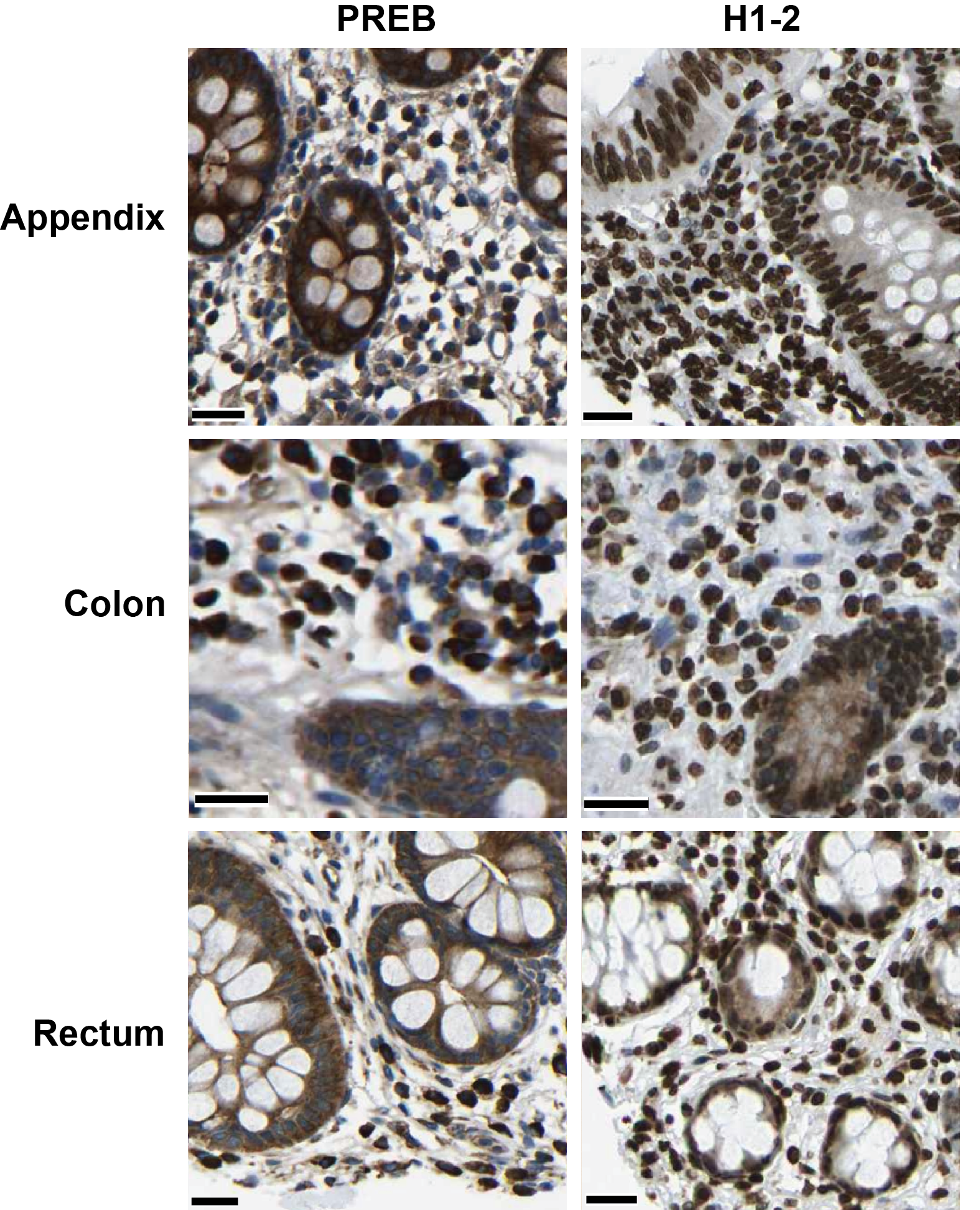


## Supplementary Figure S3. Endogenous PREB localizes to the nucleus in cells of the human appendix, colon, and rectum.

Immunohistochemistry-based protein expression profiles of PREB and H1-2 (H1.2 linker histone) in human appendix, colon, and rectum tissues (scale bar = 25 µm), showing data from the Human Protein Atlas [(77)](https://www.zotero.org/google-docs/?wRRyCX). The brown 3,3'-diaminobenzidine staining labels target proteins; the blue hematoxylin preferentially labels cell nuclei. These three profiles suggest nuclear localization of PREB in many cells in tissue sections of human appendix, colon, and rectum, but with some variation in localization across specific cell types. In comparison, the histone H1-2 exhibits nuclear localization in almost all cells in tissue-matched sections.


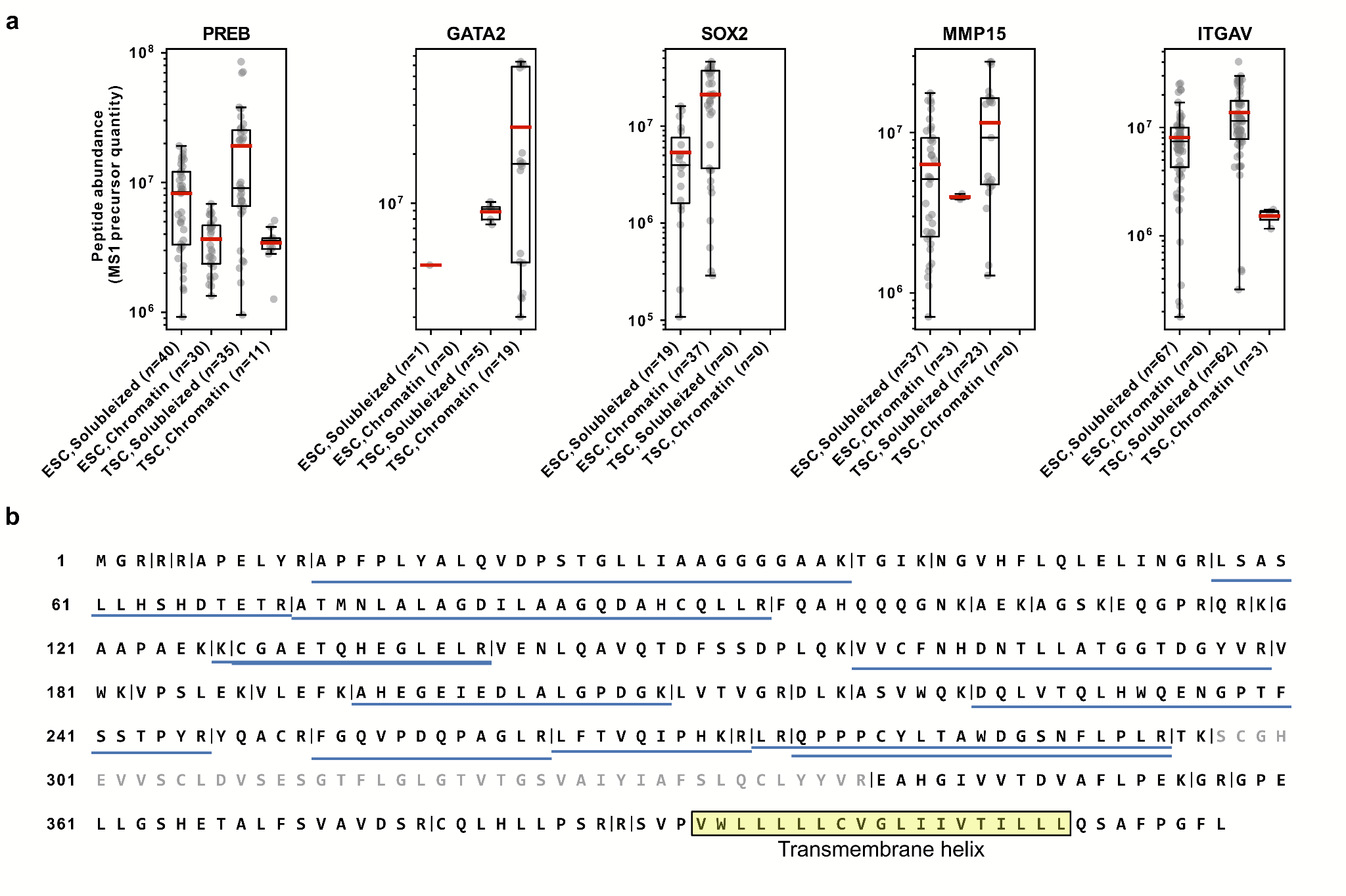


## Supplementary Figure S4. Chromatin association of endogenous PREB in human embryonic stem cells and trophoblast stem cells

(**a**) Endogenous PREB is detected in the detergent-insoluble chromatin fractions and the detergent-solubilized whole cell extract of both human embryonic stem cells (ESCs) and trophoblast stem cells (TSCs). For comparison, abundances are plotted for two cell-type-specific transcription factors (GATA2 and SOX2) and two single-pass transmembrane cell-surface proteins, the matrix metalloprotease MMP15 and integrin alpha-V (ITGAV). Abundances are quantified per peptide detected by DIA mass spectrometry across two biological replicates. Box plots indicate Q1, median, and Q3 +/- 1 IQR of measured peptide abundances (gray circles; means shown in red), with numbers of independent peptide abundance measurements indicated in parentheses. (**b**) 12 unique PREB tryptic peptides were observed across ESC and TSC samples (vertical ticks indicate tryptic cut sites, underlines indicate observed peptides), covering 62% of the amino acids from residues 1-294; crucially, no peptides from the C-terminal end were detected. It should be noted that residues 297-339 (gray) form a single tryptic peptide that exceeds the detectable mass/charge range (400-900 m/z, +2 ≤ z ≤ +4) and is not expected to be observed.


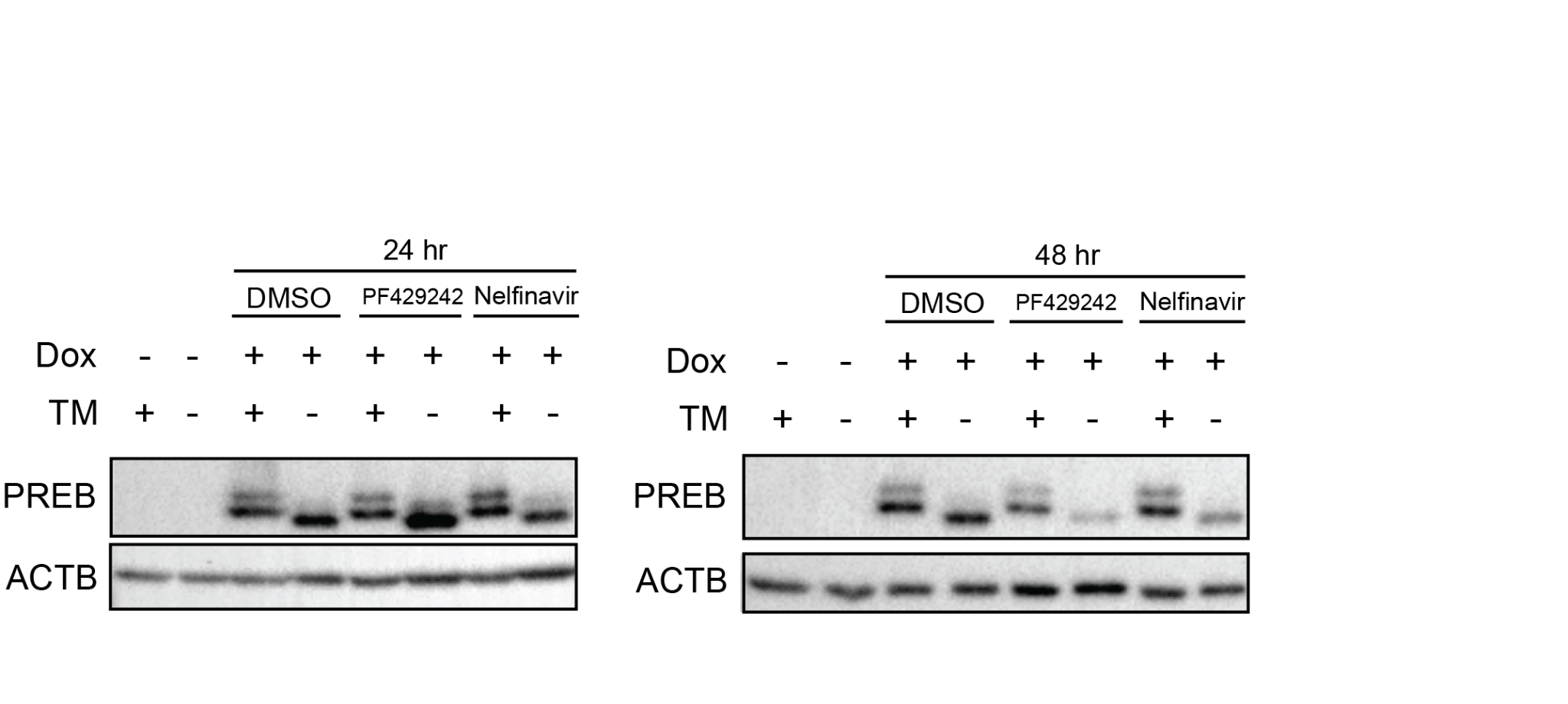


## Supplementary Figure S5. S1P and S2P inhibitors do not block the cleavage of PREB in mouse embryonic stem cells

Western blot results of PREB- and PREBΔTM-expressing mouse embryonic stem cells after applying S1P and S2P inhibitors for 24 and 48 hours. PREB clones retain two bands after inhibitor treatment, indicating that the two bands do not derive from S1P or S2P protease cleavage activity.

# Supplementary Tables

## Supplementary Table S1. Sensitivity of DeepTMHMM and TMbed for transmembrane proteins from the Protein Data Bank of TM proteins database

|  | DeepTMHMM | TMbed |
| --- | --- | --- |
| Alpha helix  (33,241 entries) | 94.79%  (31,510 identified) | 98.27%  (32,666 identified) |
| Beta strand  (1,245 entries) | 64.74%  (806 identified) | 83.37%  (1,038 identified) |

## Supplementary Table S2. Primers used for cloning

| PREB/PREBΔTM Forward Primer | GACTAGCGGCCGCAATGGGTCGGCGCCGGGGTGTG |
| --- | --- |
| PREB Reverse Primer | GGTGCTAGCTTAGAGAAATCCCGGGAAGGC |
| PREBΔTM Reverse Primer | GGTGCTAGCTTAACTCCGCCGTGAAGGCAG |

##

## Supplementary Table S3. Selected 855 [domain, orthogroup] pairs and corresponding proteins

Supplementary Table S3 is available for download at <https://doi.org/10.5281/zenodo.17351617>.

##

## Supplementary Table S4. Enriched motifs of consensus PREBΔTM ChIP-seq peaks

| Rank | Motif | Name | q-value (Benjamini) |
| --- | --- | --- | --- |
| 1 | 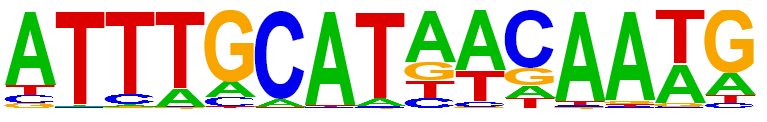 | OCT4-SOX2-TCF-NANOG(POU,Homeobox,HMG)/mES-Oct4-ChIP-Seq(GSE11431)/Homer | q < 1x10^-4^ |
| 2 | 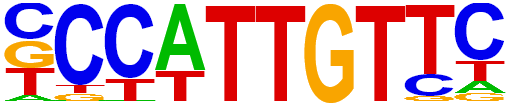 | Sox2(HMG)/mES-Sox2-ChIP-Seq(GSE11431)/Homer | q < 1x10^-4^ |
| 3 | 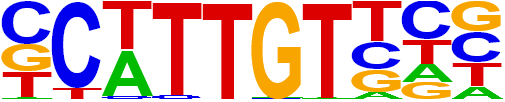 | Sox10(HMG)/SciaticNerve-Sox3-ChIP-Seq(GSE35132)/Homer | q < 1x10^-4^ |
| 4 | 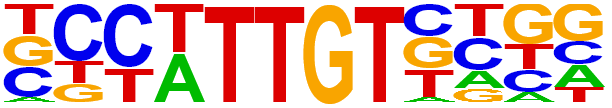 | Sox21(HMG)/ESC-SOX21-ChIP-Seq(GSE110505)/Homer | q < 1x10^-4^ |
| 5 | 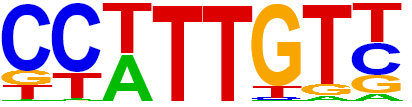 | Sox3(HMG)/NPC-Sox3-ChIP-Seq(GSE33059)/Homer | q < 1x10^-4^ |
| 6 | 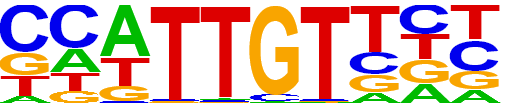 | Sox6(HMG)/Myotubes-Sox6-ChIP-Seq(GSE32627)/Homer | q < 1x10^-4^ |
| 7 | 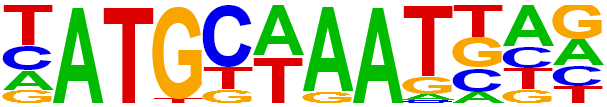 | Brn1(POU,Homeobox)/NPC-Brn1-ChIP-Seq(GSE35496)/Homer | q < 1x10^-4^ |
| 8 | 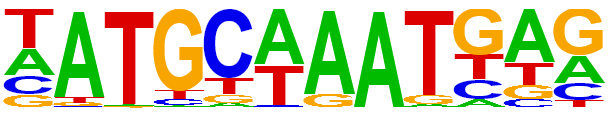 | Oct6(POU,Homeobox)/NPC-Pou3f1-ChIP-Seq(GSE35496)/Homer | q < 1x10^-4^ |
| 9 | 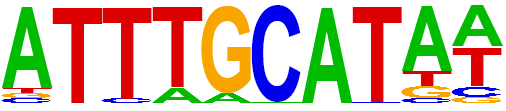 | Oct4(POU,Homeobox)/mES-Oct4-ChIP-Seq(GSE11431)/Homer | q < 1x10^-4^ |
| 10 | 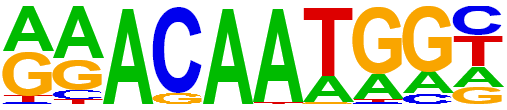 | Sox15(HMG)/CPA-Sox15-ChIP-Seq(GSE62909)/Homer | q < 1x10^-4^ |
| 11 | 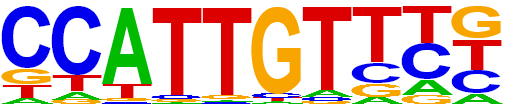 | Sox17(HMG)/Endoderm-Sox17-ChIP-Seq(GSE61475)/Homer | q < 1x10^-4^ |
| 12 | 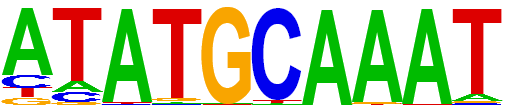 | Oct2(POU,Homeobox)/Bcell-Oct2-ChIP-Seq(GSE21512)/Homer | q < 1x10^-4^ |

##

## Supplementary Table S5. Enriched motifs in PREBΔTM-specific peaks not overlapping wildtype ESC ATAC-seq peaks

| Rank | Motif | Name | q-value (Benjamini) |
| --- | --- | --- | --- |
| 1 | 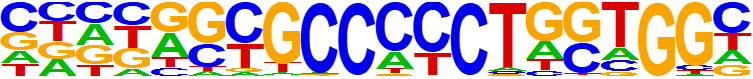 | BORIS(Zf)/K562-CTCFL-ChIP-Seq(GSE32465)/Homer | q < 1x10^-4^ |
| 2 | 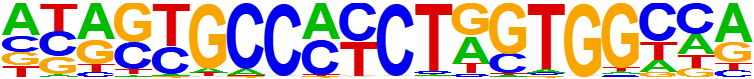 | CTCF(Zf)/CD4+-CTCF-ChIP-Seq(Barski_et_al.)/Homer | q < 1x10^-4^ |
| 3 | 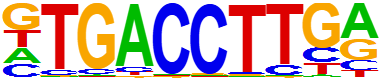 | Esrrb(NR)/mES-Esrrb-ChIP-Seq(GSE11431)/Homer | q < 1x10^-4^ |
| 4 | 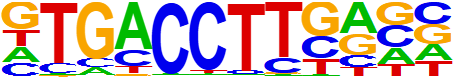 | ERRg(NR)/Kidney-ESRRG-ChIP-Seq(GSE104905)/Homer | q < 1x10^-4^ |
| 5 | 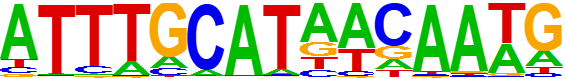 | OCT4-SOX2-TCF-NANOG(POU,Homeobox,HMG)/mES-Oct4-ChIP-Seq(GSE11431)/Homer | q < 1x10^-4^ |
| 6 | 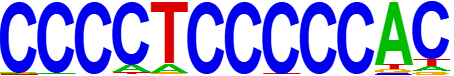 | Zfp281(Zf)/ES-Zfp281-ChIP-Seq(GSE81042)/Homer | q < 1x10^-4^ |
| 7 | 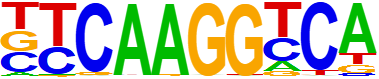 | Nr5a2(NR)/mES-Nr5a2-ChIP-Seq(GSE19019)/Homer | 1 x 10^-4^ |
| 8 | 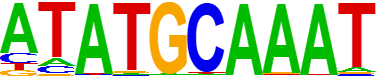 | Oct2(POU,Homeobox)/Bcell-Oct2-ChIP-Seq(GSE21512)/Homer | 1 x 10^-4^ |
| 9 | 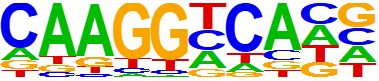 | SF1(NR)/H295R-Nr5a1-ChIP-Seq(GSE44220)/Homer | 1 x 10^-4^ |
| 10 | 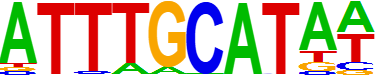 | Oct4(POU,Homeobox)/mES-Oct4-ChIP-Seq(GSE11431)/Homer | 2 x 10^-4^ |
| 11 | 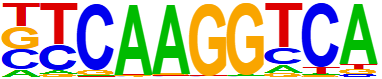 | Nr5a2(NR)/Pancreas-LRH1-ChIP-Seq(GSE34295)/Homer | 4 x 10^-4^ |
| 12 | 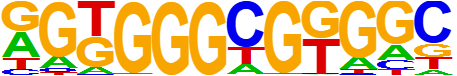 | KLF14(Zf)/HEK293-KLF14.GFP-ChIP-Seq(GSE58341)/Homer | 5.6 x 10^-3^ |

## 
